# Genetic Diversity of Sodefrin-Variant Pheromones and Pheromone Responsiveness in Subspecies of the Japanese Sword-Tail Newt *Cynops ensicauda*

**DOI:** 10.3390/ani15070947

**Published:** 2025-03-26

**Authors:** Tomoaki Nakada, Fumiyo Toyoda, Atsushi Tominaga, Koji Mochida, Makoto Yokosuka, Sakae Kikuyama

**Affiliations:** 1 Department of Veterinary Medicine, Faculty of Veterinary Medicine, Nippon Veterinary and Life Science University, Tokyo 180-8602, Japan; mayokosuka@nvlu.ac.jp; 2 Department of Physiology, Nara Medical University, Nara 634-8521, Japan; tfumiyo@naramed-u.ac.jp; 3 Department of Natural Sciences, Faculty of Education, University of the Ryukyus, Okinawa 903-0213, Japan; tominaga@edu.u-ryukyu.ac.jp; 4 Department of Biology, Keio University, Yokohama 108-8345, Japan; mochida_koji@nias.ac.jp; 5 Department of Biology, Faculty of Education and Integrated Sciences, Waseda University, Tokyo 169-8050, Japan

**Keywords:** sodefrin variants, geographic variation, peptide pheromone, *Cynops* newts

## Abstract

Experiments were conducted to examine whether the abdominal glands of *Cynops ensicauda* subspecies (*C. e. ensicauda* and *C. e. popei)* express different mRNAs for sodefrin-like female-attracting pheromone precursors. A precursor mRNA encoding a novel sex pheromone candidate, [Gln^8^]sodefrin, was widely detected in the abdominal glands of both of *C. ensicauda* subspecies. Female partners exhibited a considerable preference for synthetic [Gln^8^]sodefrin. Thus, [Gln^8^]sodefrin was designated caudarin. In *C. e. ensicauda*, the abdominal glands of males expressed sodefrin precursor mRNA as well, and sodefrin was also effective in attracting female partners. In addition to caudarin precursor mRNA, the abdominal glands of *C. e. popei* males expressed silefrin ([Leu^3^, Gln^8^]sodefrin) precursor mRNA, as reported previously. Moreover, imorin, a male-attracting pheromone previously identified in *C. pyrrhogaster* females, was effective in attracting *C. e. ensicauda* males but not males of *C. e. popei*. Molecular phylogenetic analysis indicated that trans-specific possession of sodefrin genes was likely because of gene occurrence preceding speciation of *C. ensicauda* and *C. pyrrhogaster*, and that the additional occurrence of silefrin in *C. e. popei* possibly resulted from duplication of the caudarin gene. Differences in pheromone components among *Cynops* species and subspecies reflect the speciation route estimated by previous molecular genetic analyses.

## Simple Summary

*Cynops* newts inhabiting the Japanese archipelago have been classified into two species, namely, *Cynops pyrrhogaster* and *C. ensicauda*, with the latter being divided into two subspecies, namely, *C. ensicauda ensicauda* and *C. e. popei*. Experiments were conducted to determine whether mRNA expression of a sodefrin-like female attractant pheromone precursor is differently expressed in the abdominal glands of these subspecies. The results indicate that the precursor mRNA encoding [Gln^8^]sodefrin is widely detected in the abdominal glands of both subspecies. Preference tests demonstrated that [Gln^8^]sodefrin attracts conspecific females. This novel pheromone was named caudarin. Modification of the pheromone molecules was discussed in consideration of the geographical and chronological location of the newts’ habitat.

## 1. Introduction

In urodeles, sex pheromones play an important role by allowing both sexes to find a partner and maintain reproductive interactions, proceeding into courtship behavior that ends in successful fertilization. Accordingly, it is understood that structural changes of pheromones toward more effective molecules affect subsequent reproductive success and may even cause reproductive isolation and speciation [1]. Since sodefrin (Ser-Ile-Pro-Ser-Lys-Asp-Ala-Leu-Leu-Lys) emerged as the first pheromone to be identified in amphibians and the first peptide pheromone in vertebrates [2], the existence of several peptidic [3,4,5] and proteinaceous [6] pheromones in urodeles and even in an anuran species [7] has been elucidated. Sodefrin is generated in the abdominal gland of the male red-bellied newt *Cynops pyrrhogaster*, which inhabits the Shikoku and Kyushu islands as well as the main island of Japan. It is delivered through the male’s tail-fanning behavior, exerting a potent female-attracting activity toward sexually mature conspecific females at the initial stage of courtship behavior [2], and elicits a neural response in the sensory cells of the female vomeronasal organ [8,9,10]. Conversely, males of the congeneric newt *C. ensicauda popei* inhabiting Okinawa island were revealed to possess the pheromone silefrin, whose sequence differs from that of sodefrin by two amino acid residues. The effect of both pheromones appears to be species-specific. Sodefrin attracts *C. pyrrhogaster* females exclusively, whereas silefrin attracts only *C. ensicauda* females [3,11]. The primary structures of the precursor proteins of sodefrin and silefrin have been predicted from the nucleotide sequence of cloned cDNAs [11].

The sodefrin precursor protein comprises 189 amino acid residues, and the sodefrin sequence (residues 177–186) is located close to the COOH-terminus of the protein [11]. The existence of enzymes that cleave the Arg^176^-Ser^177^ and Lys^186^-Ile^187^ bonds to generate sodefrin from the precursor was demonstrated in the abdominal glands of sexually mature *C. pyrrhogaster* males [12]. In addition to the precursors of sodefrin and silefrin, the presence of two types of sodefrin precursor-like proteins named sodefrin precursor-like factors-alpha and beta (SPF-α and -β) has been predicted from the nucleotide sequences of mRNAs expressed in the exocrine glands of several salamanders [13,14,15,16,17,18,19]. To date, multiple SPF isoforms were found to be expressed in each species, and more than 500 SPF mRNAs have been submitted to GenBank, not only for salamanders but also for frogs [20] and even a shark (sequence accession numbers are listed in Table S1). It is notable that SPFs secreted by the mental gland in the terrestrially courting lungless salamanders (Plethodontidae) themselves function as courtship pheromones [14]. More recently, the cloacal gland in the aquatically courting newt *Lissotriton helveticus* was also shown to secrete the SPF pheromone [21].

It was previously demonstrated that genes encoding the precursor protein of the peptide pheromones and SPFs that are devoid of peptide pheromone sequences belong to the same multigene family, and that sodefrin precursors have a higher sequence similarity with the SPF-β subgroup. Thus, the β subgroup has been regarded as the genetic origin of the peptide pheromone precursor proteins [22,23]. In fact, sodefrin precursor transcripts were revealed to contain an extra 62-base insertion as compared with SPF transcripts; this insertion causes a frameshift in protein translation, generating the sodefrin sequence and enzymatic cleavage sites for sodefrin [22]. Multiple genes encoding sodefrin or silefrin precursors and SPFs are known to be expressed in the abdominal gland of an individual *Cynops* newt as well [4,22,23,24].

Different morphological characteristics between *C. ensicauda* populations inhabiting both the Amami and Okinawa islands have been noticed. Accordingly, Inger (1947) divided them into two subspecies, namely, *C. e. ensicauda* and *C. e. popei*, respectively [25]. Recently, on the basis of analyses of mitochondrial and nuclear DNA sequences, it was confirmed that *C. ensicauda* species should be divided into two genetically distinct subspecies [26,27].

We previously demonstrated that the abdominal gland of male *C. e. popei* generates silefrin but not sodefrin [3,11]. In this study, we conducted experiments to see whether the abdominal gland of another subspecies, *C. e. ensicauda*, expresses sodefrin-like pheromone precursor proteins that differ from the precursors for silefrin, and if so, whether *C. e. ensicauda* females respond to other *Cynops* pheromones such as silefrin and sodefrin in addition to the putative *C. e. ensicauda* pheromone. Experiments were also conducted to see whether the males of the *C. ensicauda* subspecies would respond to a male-attracting pheromone, imorin, which is known to be delivered from the oviducal epithelium of sexually mature *C. pyrrhogaster* females [5]. A phylogenetic tree of the sodefrin precursor and sodefrin-variant precursor proteins was constructed, incorporating the data obtained in the present experiments in order to understand the evolutionary relationships of the pheromone molecules among *C pyrrhogaster*, *C. e. popei*, and *C. e. ensicauda*.

## 2. Materials and Methods

### 2.1. Animals

To obtain the abdominal glands for the analysis of transcripts encoding sodefrin or its variant precursors or SPF proteins, eight sexually mature male *C. e. ensicauda* and *C. e. popei* were collected during the breeding season (from January to March) in the suburbs of Amami and Okinawa islands, respectively. Similarly, eight sexually mature male *C. pyrrhogaster* newts captured during the breeding season (from March to April) in Chiba of the main island of Japan were used for transcript analysis. In addition, eight female *C. e. ensicauda* and *C. e. popei* obtained in February and eight female *C. pyrrhogaster* collected in Chiba and Nara of the main island in April and May were kept for one month or more in a laboratory vivarium and were used for testing the biological activity of the pheromones or pheromone candidate. Prior to the test, they were treated with 1 IU ovine prolactin (Sigma, St. Louis, MO, USA) and 15 IU human chorionic gonadotropin (Asuka Pharmaceutical Co., Tokyo, Japan) every 2 days for 14 days to bring them to a reproductive state, ensuring their responsiveness to the pheromonal substances, according to the previously described methods [28]. All animal experiments were conducted in accordance with the approved protocols and the Guidelines for the Care and Use of Laboratory Animals of the Steering Committees for Animal Experimentation at Nippon Veterinary and Life Science University and Nara Medical University, Japan.

### 2.2. Sequence Determination of cDNA Encoding Sodefrin or Its Variant Precursors or SPF Proteins

Total RNA was individually extracted from the abdominal glands of *C. e. ensicauda*, *C. e. popei*, and *C. pyrrhogaster* using ISOGEN (Nippon Gene, Tokyo, Japan) and reverse-transcribed with reverse transcriptase (SuperscriptIII: Invitrogen, Carlsbad, CA, USA). Amplification by PCR of the partial cDNAs, covering the whole open reading frame of sodefrin or its variant peptide precursors, was performed with 50 pmol of each synthetic degenerate primer (sense: 5′-ACCCTAYTCCTTACTCTCCTAGCA-3′; antisense: 5′-RTCCCCGCCATGTGGAAAAT-3′) with 0.5 units Pfu-X polymerase (Greiner Bio-One, Tokyo, Japan) per 20 μL reaction solution. Amplified DNA fragments were treated with *Ex Taq* DNA polymerase (Takara Bio, Shiga, Japan) in the presence of dATP to add A-overhang then ligated into a pT7-blue T vector (Novagen, Madison, WI, USA), and the plasmids were used to transform JM109 competent cells (Takara Bio). Eight clones from different colonies obtained individually were subjected to sequence analysis. Nucleotide sequences were analyzed by an ABI PRISM 3100-Avant Genetic Analyzer (Applied Biosystems, Foster City, CA, USA) or a commercial sequencing service (Eurofins Genomics, Tokyo, Japan).

### 2.3. Phylogenetic Tree Reconstruction

A total of 192 nucleotide sequences encoding sodefrin or sodefrin-variant peptide precursor proteins, obtained from 24 individuals consisting of 8 of each of the *C. e. ensicauda*, *C. e. popei*, and *C. pyrrhogaster* newts, were translated into amino acid sequences using the program Serial Cloner [version 2.6.1; Serial Basics; http://serialbasics.free.fr/Serial_Cloner.html (accessed on 17 February 2025)]. Nucleotide and predicted amino acid sequences were aligned using the multiple sequence alignment programs MAFFT [version 7.450; https://mafft.cbrc.jp/alignment/software/ (accessed on 17 February 2025)] and ClustalX (version 2.1 [29]), respectively, with default parameters, and minor refinements were made manually. Nucleotide sequences were then aligned, and the transcripts were named using a “species (species and subspecies from which the transcript was cloned)–protein (a protein encoded by the transcript)–isoform (serial number of the protein isoform)–transcript (serial number of the transcript)” manner. For example, “transcript 1 that encodes sodefrin precursor isoform 2 derived from *C. e. ensicauda*” was designated as “Cee-sodefrin02-1”. These transcripts were deposited in GenBank (12 transcripts for Cee-[Gln^8^]sodefrin: LC861867-LC861878; 8 transcripts for Cee-sodefrin: LC861879-LC861886; 10 transcripts for Cee-SPF: LC861887-LC861896; 2 transcripts for Cep-[Gln^8^]sodefrin: LC861897-LC861898; 5 transcripts for Cep-silefrin: LC 861899-LC861903; 13 transcripts for Cee-SPF: LC861904-LC861916; 14 transcripts for Cp-[Asn^10^]sodefrin: LC861917-LC861930; 19 transcripts for Cp-sodefrin: LC861931-LC861949; and 11 transcripts for Cp-SPF: LC861950-LC861960).

Together with the 192 amino acid sequences obtained in this work, 534 sequences of sodefrin precursor, sodefrin-variant precursor, or SPF proteins were downloaded from GenBank (the list of proteins used in the tree is shown in Table S1) and also used in tree reconstruction. Phylogenetic trees were generated by applying the maximum-likelihood and the neighbor-joining algorithms to the aligned sequences, using MEGAX software (version 10.2.6 [30]) with 1000 bootstrap replications, with corrections for gaps or multiple substitutions with corrections for misalignments.

### 2.4. Test for Biological Activity of Sodefrin, Sodefrin-Variant Peptides, and Imorin

Test substances, such as sodefrin, silefrin, and [Gln^8^]sodefrin, were synthesized by Eurofins Genomics (Tokyo, Japan). Determination of the female-attracting activity of these peptides was performed according to previously described methods [28]. Briefly, a plastic container (diameter 37 cm) was filled with 3000 mL of tap water, and a female newt was kept in a smaller cylinder of stainless steel mesh (15 cm in diameter) that had been placed in the center of the container. The container was divided into three sectors. A sponge block (5.6 cm × 7.3 cm × 3.4 cm) was gently placed into each sector. One block contained the test substance dissolved in 100 mL water. It has previously been confirmed that the minimum effective amount of sodefrin to attract conspecific females is 10 pmol when sponge blocks adsorbed with the pheromone are placed in a container filled with 3000 mL of water [2]. Consequently, the same amount of synthetic sodefrin-like peptide was used to evaluate the female-attracting activity. For the male attractant tripeptide, 30 pmol imorin was used because its molecular weight is about one third that of the decapeptide pheromones. In cases where this amount proved ineffective, the test substance was increased to 3–10 times the initial amount. The other two contained tap water (blank). Thirty seconds after the introduction of the sponge blocks, the inner cylinder was removed. The position of the snout of the test animal was observed, and the time spent by the snout in each sector was video-recorded for 10 min. In each series of tests, eight females were used. The time spent by the snout of the test animals in each sector was analyzed statistically by Friedman’s two-way analysis of variance, followed by the Wilcoxon matched-pairs signed-rank test.

The male-attracting peptide imorin was synthesized by Peptide Institute Inc. (Osaka, Japan). Its activity in the males of *Cynops* species and subspecies was determined according to previously described methods [5].

## 3. Results

### 3.1. Analysis of Transcripts Encoding Sodefrin Precursor, Sodefrin-Variant Precursor, and SPF Proteins

After removal of the primer sequences, 128 nucleotide sequences encoding sodefrin precursor, sodefrin-variant precursor, or SPF proteins that were obtained from mRNAs expressed in the abdominal gland of each of the eight individual *C. e. ensicauda*, *C. e. popei*, and *C. pyrrhogaster* newts were determined. The encoded proteins were categorized according to the predicted amino acid sequence similarity (Table A1 and Table A2, simplified as Table 1). A total of 41 clones consisting of 20 types of transcripts encoding 13 isoforms of sodefrin precursor or sodefrin-variant precursor proteins from *C. e. ensicauda*, as well as 14 clones consisting of 7 types of transcripts encoding 7 isoforms of sodefrin-variant precursor proteins from *C. e. popei*, were identified. In *C. e. ensicauda*, 11 clones (8 types of transcripts) encoding 5 isoforms of sodefrin precursor, 30 clones (12 types of transcripts) encoding 8 isoforms of sodefrin-variant precursor ([Gln^8^]sodefrin precursor), and 23 clones (10 types of transcripts) encoding 10 isoforms of SPF proteins were detected; no clones encoding silefrin ([Leu^3^, Gln^8^]sodefrin) precursor were found. In *C. e. popei*, 6 clones consisting of 2 types of transcripts that were encoded with 2 isoforms of [Gln^8^]sodefrin precursor, and 8 clones consisting of 5 types of transcripts that were encoded with 5 isoforms of silefrin precursor were recognized, whereas no clones encoding sodefrin were detected in this subspecies. However, 50 clones (13 types of transcripts) encoding 10 isoforms of SPF proteins were detected.

Similarly, 64 nucleotide sequences encoding sodefrin precursor, sodefrin-variant precursor, or SPF proteins that were obtained from mRNAs expressed in the abdominal glands of eight individual *C. pyrrhogaster* were analyzed. As a result, 28 clones consisting of 19 types of transcripts encoding 17 isoforms of sodefrin precursor, 24 clones (16 types of transcripts) encoding 11 isoforms of sodefrin-variant precursor ([Asn^10^]sodefrin), and 12 clones (11 types of transcripts) encoding 11 types of SPF proteins were identified. However, neither clones encoding [Gln^8^]sodefrin nor those encoding silefrin were detected in this species.

**Figure 1:**
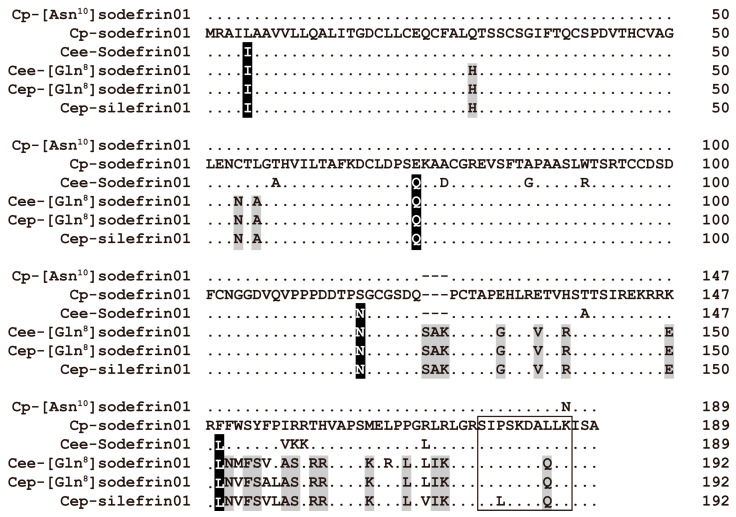
Representative amino acid sequences of sodefrin and sodefrin-variant precursor protein. The predicted amino acid sequences of the most frequently observed precursor isoforms of sodefrin and sodefrin-variant peptides is shown in an alignment format. The abbreviation at the beginning of the isoform name indicates the species or subspecies from which each sequence was obtained (Cp: *C. pyrrhogaster*; Cee: *C. e. ensicauda*; Cep: *C. e. popei*). Amino acid residues identical to those of the Cp-sodefrin01 isoform are indicated by dots. Amino acid substitutions specific to the sequences from the two subspecies of *C. ensicauda* newts and the substitutions specific to [Gln^8^]sodefrin and silefrin are shadowed in black and in grey, respectively. The ten amino acid residues boxed indicate decapeptide pheromones to be released, undergoing an enzymatic cleavage. GenBank accession number of each protein are listed in the Appendix A, Table A1 and Table A2.

In *C. e. ensicauda*, the most frequently encoded peptides were [Gln^8^]sodefrin (30 clones encoding 8 precursor isoforms) and silefrin in *C. e. popei* (8 clones encoding 5 precursor isoforms). In *C. pyrrhogaster*, along with sodefrin precursor proteins (28 clones encoding 17 precursor isoforms), proteins containing the sequence [Asn^10^]sodefrin (24 clones encoding 11 isoforms) were recognized. In the abdominal glands of both subspecies of *C. ensicauda*, a total of 28 SPF proteins were predicted from 36 transcripts derived from 99 clones (Table 1). The most frequently observed isoform in *C. e. ensicauda* was a certain type of SPF protein, designated as SPF isoform 10 (14 clones), and another type of SPF protein, designated as SPF isoform 3 (31 clones), was most common in *C. e. popei*. In *C. pyrrhogaster*, 11 types of SPF isoforms were predicted from the transcripts derived from 12 clones. The aforementioned data are summarized in Table 1; details are shown in Table A1. Structural differences in representative examples of precursor proteins of sodefrin and sodefrin-variant peptides most frequently observed in each taxon are shown in Figure 1. Among these peptides, [Gln^8^]sodefrin is a previously unknown sodefrin-variant peptide, but sodefrin, [Asn^10^]sodefrin, and silefrin all had homologous genes encoding their precursor sequences in GenBank. BLAST (version: 2.16) searches for known sequences showing the highest sequence similarity to each precursor protein at the amino acid sequence level revealed that sodefrin precursor (CAB53093.1) has 100% sequence similarity to the Cp-sodefrin precursor 01 and 99.5% sequence similarity to the Cp-[Asn^10^]sodefrin precursor 01; [Asn^10^]sodefrin precursor (AMO51436.1) has 94.7% sequence similarity to the Cee-Sodefrin precursor 01; and silefrin precursor (CAB53094.1) has 94.3% sequence similarity to the Cee-[Gln^8^]sodefrin precursor 01, 95.3% sequence similarity to Cep-[Gln^8^]sodefrin precursor 01, and 96.9% sequence similarity to Cep-silefrin precursor 01.

Several common amino acid substitutions were found among the predicted amino acid sequences from *C. ensicauda* (black-shadowed residues in Figure 1). However, many amino acid substitutions were shared among the [Gln^8^]sodefrin and silefrin precursor isoforms, including the insertion of three amino acid residues (Ser-Ala-Lys) at positions 124–126 (Figure 1). The sodefrin precursor protein isoform predicted from the mRNA expressed in *C. pyrrhogaster* (Cp-sodefrin01) showed 99.5% sequence similarity to the Cp-[Asn^10^]sodefrin 01 isoform, which is also expressed in *C. pyrrhogaster*, and showed 93.1% sequence similarity to the Cee-sodefrin01 isoform expressed in *C. e. ensicauda*. The majority of clones encoding precursor proteins of these peptides contained a predicted open reading frame of 567 bp, which encoded a precursor protein of 189 amino acid residues including a region containing hydrophobic amino acids that was presumed to be a signal peptide on the N-terminus, in addition to the sodefrin or [Asn^10^]sodefrin sequence near the C-terminus region. Similarly, the [Gln^8^]sodefrin precursor protein isoform predicted from the mRNA expressed in *C. e. popei* (Cep-[Gln^8^]sodefrin01) showed 98.4% sequence similarity to the Cep-silefrin01 isoform also expressed in *C. e. popei*, and 97.9% sequence similarity to the Cee-[Gln^8^]sodefrin01 isoform expressed in *C. e. ensicauda*. The majority of clones encoding precursor proteins of these peptides contained a predicted open reading frame of 576 bp. The predicted amino acid sequences homologous to sodefrin decapeptide were expected to be flanked by monobasic amino acid cleavage sites, arginine and lysine (Figure 1), for generating [Gln^8^]sodefrin or silefrin peptides from the precursor.

### 3.2. Phylogenetic Analysis of the Sodefrin Precursor-Related Proteins

**Figure 2:**
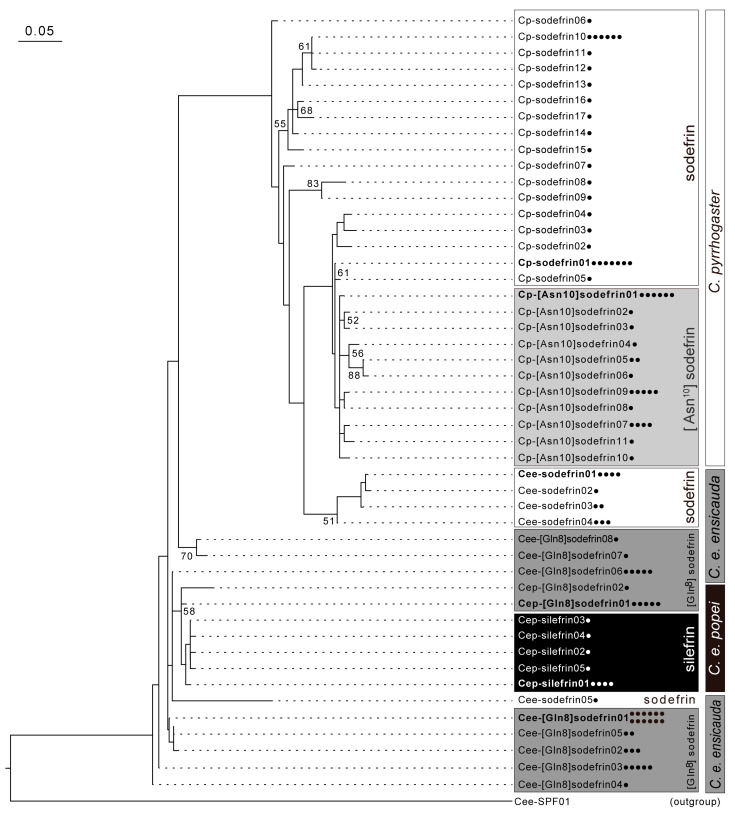
Phylogenetic tree for 50 precursor proteins containing sodefrin or sodefrin-variant sequences that were predicted from transcripts of *C. pyrrhogaster* and two subspecies of *C. ensicauda*. The molecular phylogenetic relationships of sodefrin precursor-related proteins were analyzed. One sequence of the sodefrin precursor-like factor (SPF) isoform was used as an outgroup. Amino acid sequences of 50 sodefrin and sodefrin-variant precursor isoforms were predicted from the 107 transcripts, and the sodefrin-variant precursor isoforms were divided into three types of proteins according to the [Asn^10^]sodefrin, silefrin, and [Gln^8^]sodefrin sequences they contain. Labels are designated by a “species sodefrin or sodefrin-variant sequence” manner (for example, “silefrin precursor isoform 1 derived from *C. e. popei*” was described as “Cep-silefrin01”), circles on the tip labels indicate the number of clones identified, and squared labels were used to refer to the proteins predicted from *C. pyrrhogaster*, *C. e. ensicauda*, and *C. e. popei* transcripts, respectively. The tree was reconstructed by using the maximum-likelihood method. Numbers at the nodes are bootstrap values expressed as percentages. Only bootstrap values greater than 50 are shown. The scale bar represents the number of amino acid substitutions per residue. More detailed information is available in the Appendix A, Table A1 and Table A2.

**Figure A1:**
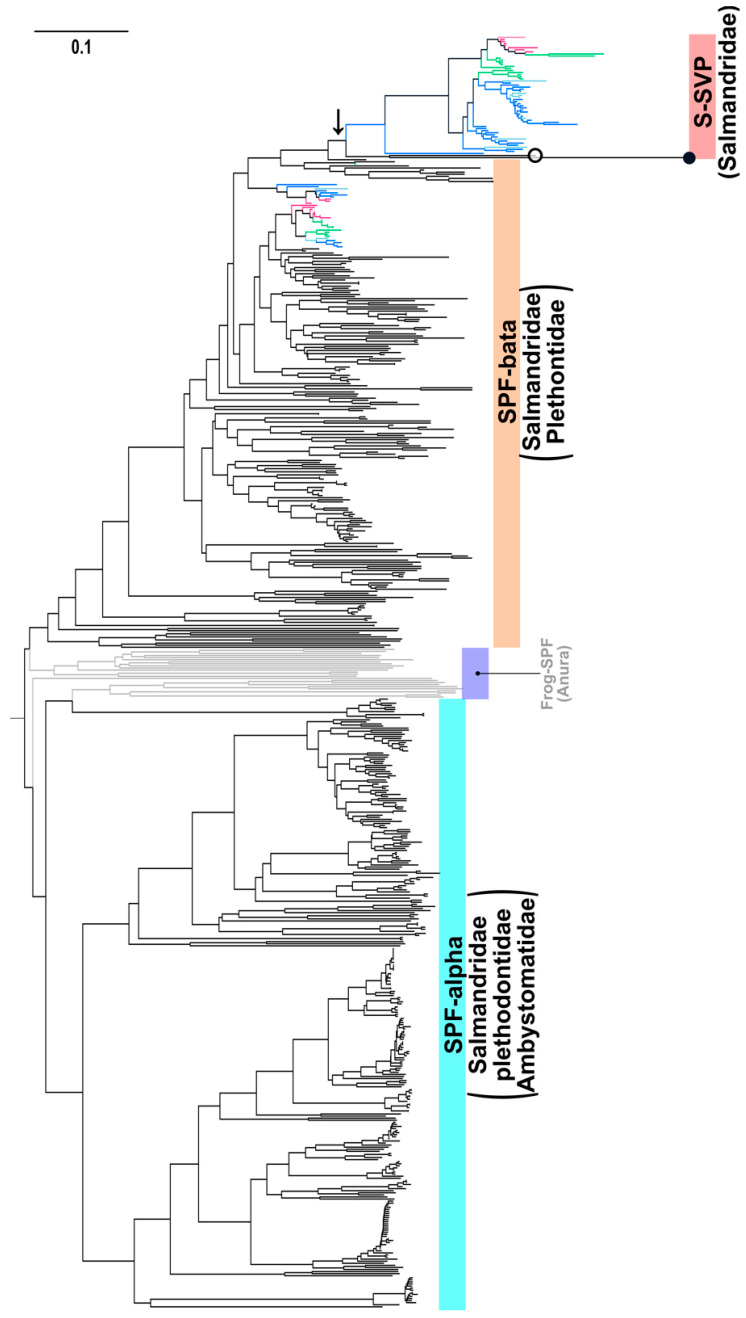
Phylogenetic tree for sodefrin, sodefrin-variant precursor proteins, and sodefrin precursor-like factor predicted from the transcripts from the abdominal glands of *C. e. ensicauda*, *C. e. popei*, and *C. pyrrhogaster*, with 534 known sequences obtained from the database. The molecular phylogenetic relationships of the newly obtained isoforms predicted from the cloned transcripts were analyzed with known sodefrin precursor-related proteins [sodefrin and sodefrin-variant precursor (S-SVP) isoforms and two subgroups (alpha and beta subgroup) of sodefrin precursor-like factor (SPF-alpha and -beta) isoforms]. It is noteworthy that the S-SVP isoforms, encompassing the cloned isoforms in this study, constitute a monophyletic group derived from a subgroup of the SPF-beta subfamily (the arrow indicates the root of the S-SVP clade). The lines of the nodes, indicating the isoforms obtained in this study, are color-coded red for *Cynops pyrrhogaster*-derived isoforms, green for *C. ensicauda ensicauda*-derived isoforms, and blue for *C. e. popei*-derived isoforms. In instances where a line corresponds to an existing *Cynops*-derived isoform within the same clade as the newly identified isoform, a lighter color was utilized to denote the species from which it was derived. Circles are employed to indicate the S-SVP isoform from *Lissotriton helveticus* (black circles) and *Triturus carnifex* (white circles), respectively. Due to the large number of sequences analyzed, the tree was reconstructed using the neighbor-joining method, with the midpoint of the longest path serving as the root. The scale bar represents the number of amino acid substitutions per residue.

**Figure A2:**
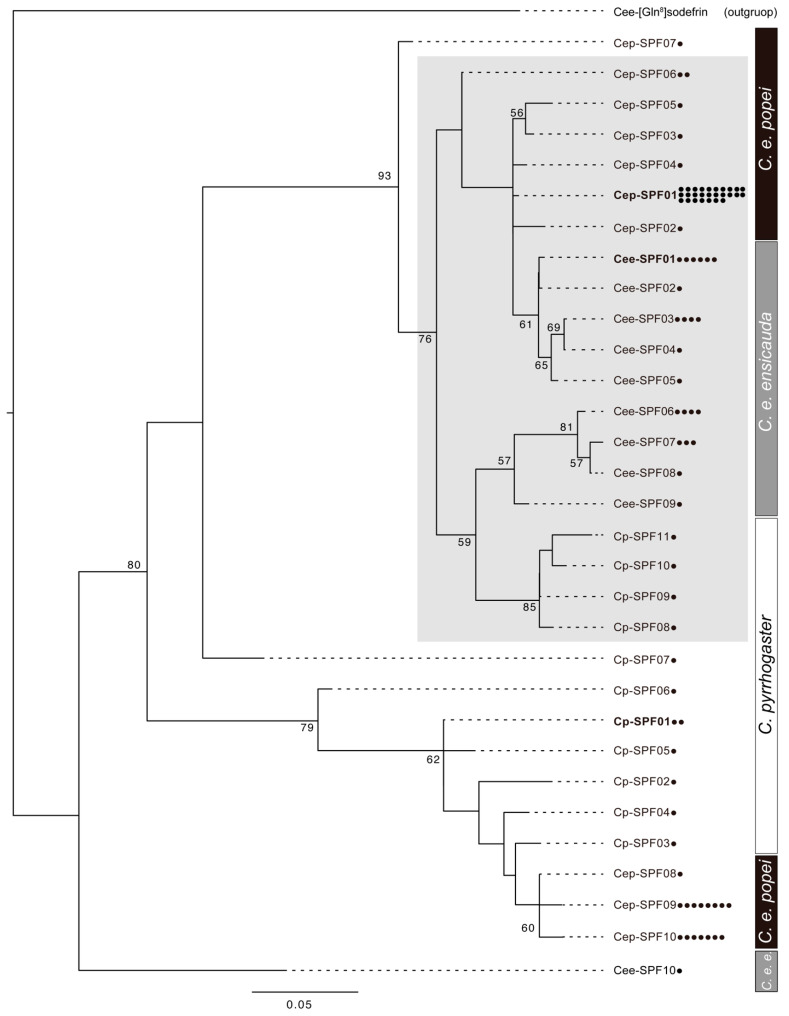
Phylogenetic tree for 31 sodefrin precursor-like factors that were predicted from transcripts of *C. pyrrhogaster* and two subspecies of *C. ensicauda*. The molecular phylogenetic relationships of sodefrin precursor-related proteins were analyzed. One sequence of the [Gln^8^]sodefrin precursor isoform was used as an outgroup. A total of 31 SPF isoforms were predicted from the 85 transcripts. Labels are designated via the “species–SPF isoform” manner (for example, the “SPF precursor isoform 1 derived from *C. e. popei*” was described as “Cep-SPF01”). The circles on the tip labels indicate the numbers of clones identified, and squared labels are used to refer to the proteins predicted from *C. pyrrhogaster*, *C. e. ensicauda*, and *C. e. popei* transcripts, respectively. The clade consisting of isoforms with the highest number of clones observed within the two subspecies of *C. ensicauda* is enclosed in the gray square. The tree was reconstructed using the maximum-likelihood method. The numbers at the nodes are bootstrap values expressed as percentages. Only bootstrap values greater than 50 are shown. The scale bar represents the number of amino acid substitutions per residue. More detailed information is available in Table A1 and Table A2.

The relationships of the sodefrin or sodefrin-variant precursor proteins predicted from the transcripts obtained from *C. e. ensicauda*, *C. e. popei*, and *C. pyrrhogaster* were inferred through phylogenetic trees constructed both with and without the 534 known sequences obtained from Genbank (Table S1). The reconstructed tree from analysis of all obtained protein sequences clearly indicated that sodefrin precursor-like proteins consisted of two types of SPF group (SPF-α and -β) in addition to a sodefrin and sodefrin-variant precursor (S-SVP) subgroup that belongs to the SPF-β subfamily. All of the proteins predicted from the transcripts obtained here from the *Cynops* newts were classified as members of the S-SVP or SPF group in the tree, and all the predicted SPF proteins obtained in this study were revealed to belong to the SPF-β subfamily (Figure A1). The molecular phylogenetic relationships of SPFs and S-SVPs obtained here were analyzed separately from the existing dataset (Figure 2 and Figure A2). In *C. ensicauda*, 20 S-SVP and 20 SPF isoforms were predicted from the 128 transcripts, and S-SVP isoforms were divided into three types of protein that were estimated to generate different peptides, such as sodefrin, silefrin, and [Gln^8^]sodefrin. Although 6 of 20 S-SVP isoforms and 7 of 20 SPF isoforms were found to be intra-subspecifically common, no isoforms were found to be shared with another subspecies (Figure 2 and Figure A2). In *C. pyrrhogaster*, 28 S-SVP isoforms and 11 SPF isoforms were predicted from the 64 transcripts, and S-SVPs were divided into two types of protein that were estimated to generate different peptides (sodefrin and [Asn^10^]sodefrin) (Table 1; Figure 2). Although 5 of the 28 S-SVP isoforms and 1 of the 11 SPF isoforms were found to be intraspecifically common, no isoforms were found to be shared with *C. ensicauda.*

### 3.3. Evaluation of the Pheromonal Activity of Sodefrin, Sodefrin Variants and Imorin

**Figure 3:**
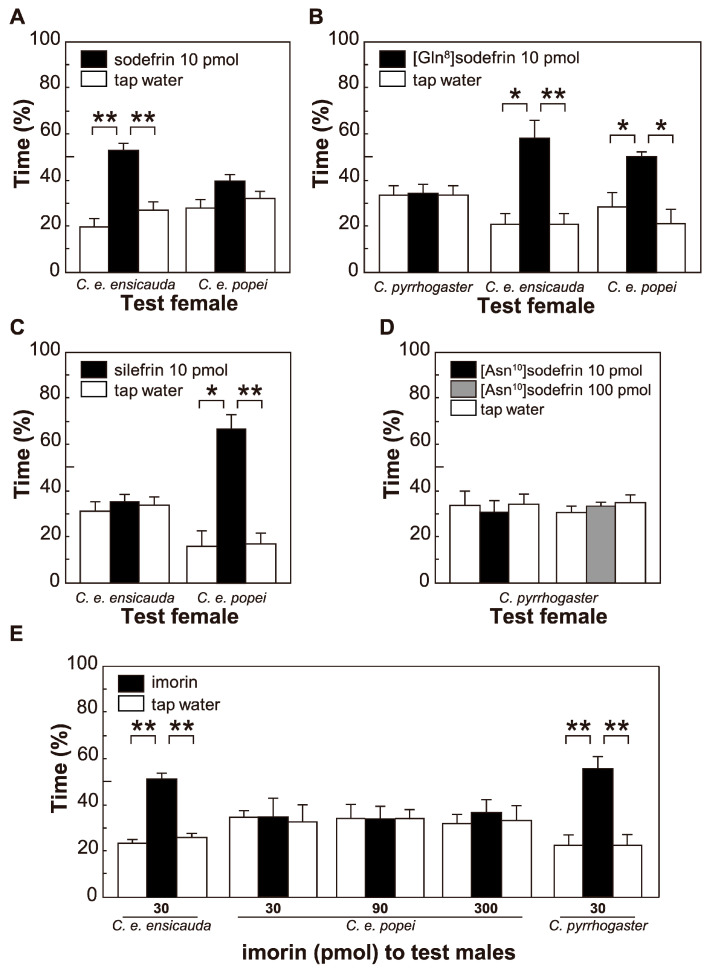
Responsiveness of *Cynops* female newts to sodefrin and its variants and of *Cynops* male to imorin. The female-attracting activities of sodefrin (**A**), [Gln^8^]sodefrin (**B**), silefrin ([Leu^3^, Gln^8^] sodefrin) (**C**), and [Asn^10^]sodefrin (**D**) were tested by using sexually mature female *Cynops* newts. Similarly, the male-attracting activity of imorin, a tripeptide pheromone released by *C. pyrrhogaster* female (**E**), was tested using sexually mature male newts. Each test was performed on different specimens (*n* = 8). Each column and vertical bar represent the mean ± SEM. (**A**) *C. e. ensicauda* females were attracted by sodefrin, but *C. e. popei* females were not (** *p* < 0.01). (**B**) *C. pyrrhogaster* females were not attracted by [Gln^8^]sodefrin, but *C. e. ensicauda* and *C. e. popei* females showed a marked preference for [Gln^8^]sodefrin (* *p* < 0.05; ** *p* < 0.01). (**C**) *C. e. popei* females, but not *C. e. ensicauda* females, were attracted by silefrin (* *p* < 0.05; ** *p* < 0.01). (**D**) *C. pyrrhogaster* females respond to neither 10 pmol nor 100 pmol [Asn^10^]sodefrin. (**E**) *C. e. ensicauda* males, as well as *C. pyrrhogaster* males, but not *C. e. popei* males, were attracted to imorin (** *p* < 0.01). Behavioral tests of sodefrin and silefrin with *C. pyrrhogaster* females were skipped because it has been established that sodefrin, not silefrin, is effective in attracting the females of this species [11]. [Asn^10^] sodefrin was tested tentatively with *C. pyrrhogaster* females because it is absent in the products of the abdominal gland [12,27].

Behavioral responses to sodefrin, silefrin, [Gln^8^]sodefrin, and [Asn^10^]sodefrin of female newts belonging to *C. e. ensicauda*, *C. e. popei*, and *C. pyrrhogaster* are shown in Figure 3. When sodefrin was applied to *C. ensicauda* females, *C. e. ensicauda* females were attracted by sodefrin, whereas *C. e. popei* females were not (Figure 3A). When [Gln^8^]sodefrin was tested with the females of the two subspecies, females belonging to both *C. ensicauda* subspecies were attracted by it, whereas *C. pyrrhogaster* females were not (Figure 3B). When silefrin was tested with females of the two subspecies of *C. ensicauda*, *C. e. popei* females were attracted by the peptide, whereas *C. e. ensicauda* females were not (Figure 3C). It was also confirmed that *C. pyrrhogaster* females were attracted by neither 10 pmol nor 100 pmol [Asn^10^]sodefrin (Figure 3D).

Subsequently, sexually mature *C. pyrrhogaster*, *C. e. ensicauda*, and *C. e. popei* males were tested with imorin for its male-attracting activity. As shown in Figure 3E, both *C. pyrrhogaster* and *C. e. ensicauda* males responded positively to 30 pmol imorin. However, *C. e. popei* males were not attracted, even with 300 pmol imorin.

### 3.4. Obtaining and Organizing the Amino Acid Sequences of Known Sodefrin or Sodefrin-Variant Precursor and SPF Proteins

Five hundred and thirty-four amino acid sequences of sodefrin precursor and sodefrin-variant precursor proteins were downloaded from GenBank (Table S1). Ten transcripts that had been identified from three species of Salamandridae newts are available in the GenBank database. Three of the ten transcripts (GenBank accession numbers KM463792.1, KU213617.1, and KU213619.1) were registered as SPF transcripts, although their sequences differed from the other SPF precursor transcripts by the presence of a 62-base insertion causing a frameshift to generate the sodefrin-variant peptide sequence ([Asn^10^]sodefrin: Ser-Ile-Pro-Ser-Lys-Asp-Ala-Leu-Leu-**Asn**), of which cDNA clones were previously isolated from the mRNAs expressed in the abdominal gland of *C. pyrrhogaster* [24]. Because the tridecapeptide COOH-terminally extended with [Asn^10^]sodefrin-ISA peptide (Ser-Ile-Pro-Ser-Lys-Asp-Ala-Leu-Leu-**Asn**-**Ile**-**Ser**-**Ala**) was isolated from the abdominal glands of the newts in the previous study, we categorized these three transcripts into the sodefrin-variant peptide group rather than into the SPF, the tentative proteinaceous pheromone group [13]. In addition to the [Asn^10^]sodefrin transcripts, two transcripts lacking the 62-base insertion (AF446080.1 from *Triturus carnifex* and KJ402351.1 from *Lissotriton helveticus*) were also classified as sodefrin variant transcripts because of a frameshift that generated sequences homologous to sodefrin (**Cys**-**Thr**-Pro-Ser-Lys-Asp-Ala-Leu-Leu-**Glu** and **His**-Ile-**Ala**-**Arg**-Lys-Asp-Ala-Leu-**Pro**-Lys, respectively). As shown in Table S1, 524 amphibian SPF transcripts are available in GenBank. In urodeles, 500 SPF transcripts have been registered and identified mainly in Salamandroidea (300, 13, and 187 transcripts from 10 salamandrid newts, 1 ambystomatid salamander (*Ambystoma mexicanum)*, and 30 plethodontid salamanders, respectively.). In anurans, 24 SPF transcripts have been registered in the database (18 transcripts from four hylid frogs, 2 transcripts from two nyctibatrachid frogs, 1 transcript from one pipid frog, and 3 transcripts from one ranid frog).

## 4. Discussion

In the present study, we performed analyses of transcripts encoding sodefrin precursor, sodefrin-variant precursor, and SPF proteins using the abdominal glands of male *C. pyrrhogaster* and two *C. ensicauda* subspecies, *C. e. ensicauda* and *C e. popei.* Our results revealed that the abdominal glands of both *C. ensicauda* subspecies possess a novel sodefrin-variant pheromone, [Gln^8^]sodefrin. Because this decapeptide exerts considerable female-attracting activity, this pheromone was designated caudarin (cauda- from the species name “ensicauda”, and -rin from sodefrin).

When the presence of the peptide pheromone silefrin was reported in the abdominal gland of *C. e. popei* from Okinawa, there was no clear explanation for the origin of this “sodefrin-like” pheromone [11]. Information concerning sodefrin-variant peptides in the abdominal gland of *C. e. ensicauda* obtained in the present study enables the explanation that silefrin diverged from caudarin as a result of a genetic mutation in a duplicated caudarin gene encoding the third amino acid residue, leucine instead of proline. It was also suggested that the precursor proteins of the novel pheromone candidate caudarin ([Gln^8^]sodefrin) and silefrin ([Leu^3^, Gln^8^]sodefrin) share a common protein ancestor that appeared more recently compared with that of S-SVP isoforms, and that predicted sodefrin precursor proteins expressed in *C. e. ensicauda* have a closer phylogenetic relationship with those expressed in *C. pyrrhogaster* as compared with any other precursors expressed in *C. e. ensicauda*. Similarly, we postulate that the caudarin gene originated from the sodefrin gene, and that the sodefrin gene disappeared from the abdominal gland of *C. e. popei* during the course of allopatric separation. As shown in the molecular phylogenetic analysis in Figure 3, the precursors of Cee-sodefrin have a closer relationship in sequence similarity with the precursors of Cp-sodefrin than those of the S-SVPs found in *C. e. ensicauda*. This supports the assumption that the ancestral sodefrin gene common to the Cp-sodefrin and Cee-sodefrin genes existed prior to the divergence of *C. pyrrhogaster* and *C. ensicauda*—the earliest divergence analyzed in this study—and may be the root gene for several of the S-SVP genes that arose from the gene duplications and were found in the subsequently emerging taxa. Similarly, the presence of the ancestral caudarin gene common to the Cee-caudarin and Cep-caudarin genes in the common ancestor of both subspecies prior to the divergence of the two *C. ensicauda* subspecies supports the assumption that the silefrin gene may have originated from the ancestral caudarin gene.

**Figure 4:**
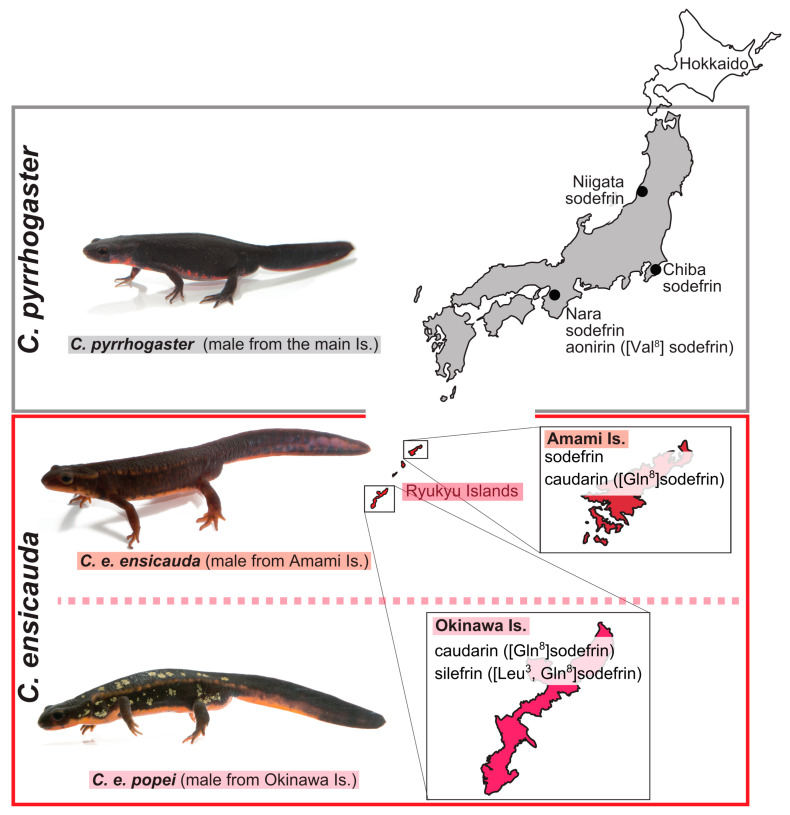
Geographic distribution and male pheromone variants of *Cynops* species in Japan. Red-bellied newts, *C. pyrrhogaster*, are found throughout the main islands except for the Hokkaido (blank) and Ryukyu islands (Is) (red). Although mRNAs coding a sex pheromone peptide (sodefrin) have been confirmed to be invariably expressed in the abdominal glands of the male individuals captured in three different areas of the main island of Japan (Niigata, Chiba, and Nara), mRNA and the translated peptide of a region-specific sodefrin variant, aonirin, have been recognized in the abdominal glands of the males captured in Nara, together with those of sodefrin [4]. Sword-tailed newts, *C. ensicauda*, inhabiting Ryukyu islands, are classified into two subspecies, namely, *C. e. popei* in Okinawa island and *C. e. ensicauda* in Amami island. The male of *C. e. popei*, inhabiting Okinawa island, has been known to produce the pheromone silefrin, which is comparable to sodefrin in *C. pyrrhogaster* [11].

As shown in Table 1, the presence of [Asn^10^]sodefrin transcripts was recognized in seven out of eight specimens of *C. pyrrhogaster.* According to our previous work [24], we could not find [Asn^10^]sodefrin (SIPSKDALLN) as a peptide form, but [Asn^10^]sodefrin C-terminally extended with three amino acid residues (SIPSKDALLN**ISA**) was detected among the fractions chromatographically separated from the abdominal gland extract. A preference test of [Asn^10^]sodefrin-ISA revealed that it had no female-attracting activity [24,27]. In the present experiment, [Asn^10^]sodefrin did not exhibit pheromonal activity (Figure 3D). Thus, we conclude that in *C. pyrrhogaster*, only sodefrin serves as a courtship pheromone, except for the case of aonirin, which is specifically involved in spousal communication in newts from the Nara region [3] (see Figure 4), and the [Asn^10^]sodefrin gene disappeared from *C. ensicauda*.

Additionally, two transcripts encoding sodefrin and sodefrin variants, which are active in terms of attracting conspecific females, were recognized in the abdominal gland of *C. ensicauda* subspecies (Table 1). Although both precursor protein mRNAs (i.e., sodefrin and caudarin in *C. e. ensicauda*; caudarin and silefrin in *C. e. popei*) were confirmed to be expressed, few specimens were found to possess both transcripts. We presume that once speciation of *C. e. ensicauda* and *C. e. popei* is complete, they will possess exclusively either caudarin or silefrin, provided that females exhibit stronger preferences for either. It is notable that pheromonal peptides, such as sodefrin, caudarin, and silefrin, that were identified in the males of the *Cynops* species and subspecies specifically attract their own groups’ female partners, and females are not attracted to sex pheromones that are not possessed by the males in their population. This means that receptors for each pheromone were newly equipped within the females’ vomeronasal organs during speciation and subspeciation, losing the receptors for pheromones that were once possessed but then lost by the male partner.

Imorin is a male-attracting pheromone identified in the oviducal epithelium of *C. pyrrhogaster* [5]. Although it has been confirmed that this tripeptide pheromone is effective in attracting sexually mature conspecific males, our results show that it attracts *C. e. ensicauda* males but not those of *C. e. popei* (Figure 3E); that is, the species or subspecies for which imorin was found to be effective were those for which sodefrin was effective. We thus speculate that the common ancestor of *C. pyrrhogaster* and *C. ensicauda* maintained spousal communication via sodefrin (male pheromone) and imorin (female pheromone), which have been shown to be used conservatively in the two species even today. It remains to be investigated, however, whether *C. e. popei* females utilize a male-attracting pheromone comparable to imorin.

As stated by Houck et al. [14], the existence of precursor proteins devoid of pheromonal peptide sequences generated as a result of gene duplication has been documented. Furthermore, the extract of the mental gland of the terrestrially courting salamanders (Plethodontidae) has been shown to exert pheromonal activity. These novel proteins were designated sodefrin precursor-like factors (SPFs) due to their sequence similarity with the sodefrin precursor protein. A comparison of the transcripts of the SPF family proteins and the sodefrin precursor protein revealed that the latter contains an additional 62-base insertion. This insertion is hypothesized to have induced a frame shift, resulting in the formation of the sodefrin peptide sequence and the subsequent emergence of enzymatic cleavage sites [22]. Nevertheless, the presence of transcripts encoding precursors with sodefrin-homologous sequences has been documented in *Triturus carnifex* and *Lissotriton helveticus*, the only taxon confirmed to utilize decapeptide pheromones is the genus *Cynops* so far; and the emergence of S-SVPs is estimated to have occurred after 34.3–26.5 MYA, coinciding with the divergence of the Asian newts that include this genus [22,31].

To date, the significance of SPF as a sex pheromone in *Cynops* newts is not well understood, but SPF transcripts have been cloned relatively frequently, especially in *C. ensicauda* (Table 1). Molecular phylogenetic analysis of the SPF proteins observed in this study identified a particular group of homologous SPF genes across the subspecies (the clade enclosed by the gray square in Figure A2). Because SPF may act as a sex pheromone in other species [14], these inter-subspecifically conserved SPFs expressed in *C. ensicauda* might also exhibit some pheromonal activity during courtship behavior.

According to previous analyses [26,31], genetic isolation between *C. pyrrhogaster* and *C. ensicauda* is estimated to have been established 13.75–12.40 million years ago (MYA), followed by the divergence between *C. e. ensicauda* and *C. e. popei* at 5.21 MYA. Therefore, the origin of reproductive communication by sodefrin and imorin, which is common to both species, must have occurred before the *C. pyrrhogaster*/*C. ensicauda* divergence. The origin of caudarin, which is common to the two subspecies of *C. ensicauda* but absent in *C. pyrrhogaster*, can be dated to 8.54–7.19 MYA, i.e., between the divergence of the two species and the divergence of the *C. ensicauda* subspecies. Similarly, silefrin, which is expressed only in *C. e. popei* males, is assumed to have acquired its function as a sex pheromone after the divergence of the two *C. ensicauda* subspecies (5.21 MYA). Focusing on the distribution of conserved amino acid residues among the sequences of precursor proteins of sodefrin, caudarin, and silefrin (Figure 1), it is obvious that the genes for caudarin and silefrin precursors were derived from those for sodefrin and caudarin precursors, respectively. Thus, accompanying the generation of caudarin and silefrin genes, in addition to the loss of [Asn^10^]sodefrin and sodefrin genes, speciation and subspeciation occurred successively. It is notable, as has been pointed out previously [26,31], that time periods of 12.40 and 5.21 million years were required for speciation and subspeciation, respectively.

A sex pheromone such as sodefrin is one of the critical factors for reproductive success and should be under high selective pressure for resistance to loss-of-function mutations. Conversely, the acquisition of a novel pheromone that is preferred by mating partners over an existing pheromone used by reproductive competitors would be an evolutionarily adaptive event. The multiplexing and diversity of male sex pheromone genes in newts demonstrated in this study may provide a solution to this conflict by not only conservatively maintaining the existing sex pheromone system (e.g., sodefrin in *C. e. ensicauda*) but also by exploring the co-evolution of the partner’s response system to acquire a new and more effective pheromonal communication system (e.g., silefrin in *C. e. popei*). In fact, such multiplexing has been observed in other vertebrates (e.g., main urinary proteins and exocrine gland-secreting peptides in rodents [32,33]), as well as in amphibians (e.g., SPFs, plethodontid modulating factors [13,34], and plethodontid receptivity factors [6,35]).

## 5. Conclusions

In conclusion, the present experiment provided evidence for the two *Cynops* subspecies, *C. ensicauda ensicauda* and *C. e. popei*, inhabiting the Amami and Okinawa Islands in isolation, having come to possess different courtship pheromones, namely, caudarin and silefrin, accompanying the loss of the [Asn^10^]sodefrin precursor gene and the acquisition of the caudarin precursor gene in the course of speciation, and similarly, the loss of the sodefrin precursor gene along with the acquisition of the silefrin precursor gene in the course of subspeciation (Figure 4).

## Figures and Tables

**Table 1:** List of sodefrin precursor-related proteins expressed in the abdominal gland of *Cynops* newts.

	Sodefrin or Sodefrin Variant Sequences to Be Contained	Amino Acid Sequences of Sodefrin or Sodefrin Variants	Number of Amino Acid Residues	Number of Isoforms	Total Number of Clones	Number of Clones per Individual **							
						A	B	C	D	E	F	G	H
*Cynops* *ensicauda* *ensicauda*	Presence	SIPSKDALLK (sodefrin)	189	5	11	6	3	1	1				
		SIPSKDA**Q**LK ([Gln^8^]sodefrin)	102–192	8	30		3	4	4	6	5	4	4
	Absence *		163–179	10	23	2	2	3	3	2	3	4	4
*Cynops* *ensicauda* *popei*	Presence	SIPSKDA**Q**LK ([Gln^8^]sodefrin)	192–205	2	6					3	1	1	1
		SI**L**SKDA**Q**LK (silefrin)	173–192	5	8	4	2	1	1				
	Absence		129–179	10	50	4	6	7	7	5	7	7	7
*Cynops* *pyrrhogaster*	Presence	SIPSKDALLK (sodefrin)	150–189	17	28	4	5	1	3	5	3	4	3
		SIPSKDALL**N** ([Asn^10^]sodefrin)	173–189	11	24		3	4	4	3	4	3	3
	Absence		140–179	11	12	4		3	1		1	1	2

* Sodefrin precursor-related proteins that are devoid of sodefrin or sodefrin-variant sequences are designated as sodefrin precursor-like factors (SPFs) [13,14]. ** Eight individual male newts subjected to analyses were tentatively described alphabetically.

## Data Availability

The original sequence data presented in the study are openly available in NCBI GenBank at https://www.ncbi.nlm.nih.gov/genbank/, accessed on 17 February 2025.

## Supplementary Materials

The following supporting information can be downloaded at https://www.mdpi.com/article/10.3390/ani15070947/s1: Table S1: List of the predicted sodefrin and its variant precursor (S-SVP) and sodefrin precursor-like factor (SPF).

## Appendix A

**Table A1:** List of transcripts encoding sodefrin and sodefrin-variant precursor (S-SVP) and sodefrin precursor-like factor (SPF) that were found to be expressed in either of the *Cynops ensicauda* subspecies.

Group	Abbreviated Transcript Name	Accession	mRNA Length (Base)	Number of Amino Acid Residues	Total Number of Clones	Number of Clones per Individual															
						*Cynops ensicauda* *ensicauda*								*C. e. popei*							
						A	B	C	D	E	F	G	H	A	B	C	D	E	F	G	H
**Sodefrin and Sodefrin-variant Precursor (S-SVP)**	**S-SVP subgroup**		**316** **–625**	**102** **–205**	**55**	**6**	**6**	**5**	**5**	**6**	**5**	**4**	**4**	**4**	**2**	**1**	**1**	**3**	**1**	**1**	**1**
	**Sodefrin precursor**		**577**	**189**	**11**	**6**	**3**	**1**	**1**												
	Cee-sodefrin01-1	LC861879	577	189	3	3															
	Cee-sodefrin01-2	LC861880	577	189	1	1															
	Cee-sodefrin02-1	LC861881	577	189	1		1														
	Cee-sodefrin03-1	LC861882	577	189	1			1													
	Cee-sodefrin03-2	LC861883	577	189	1				1												
	Cee-sodefrin04-1	LC861884	577	189	2		2														
	Cee-sodefrin04-2	LC861885	577	189	1	1															
	Cee-sodefrin05-1	LC861886	577	189	1	1															
	**[Gln^8^]sodefrin precursor**		**316** **–625**	**102** **–205**	**36**		**3**	**4**	**4**	**6**	**5**	**4**	**4**					**3**	**1**	**1**	**1**
	Cee-[Gln^8^]sodefrin01-1	LC861867	586	192	10		3	2	4				1								
	Cee-[Gln^8^]sodefrin01-2	LC861868	586	192	2					2											
	Cee-[Gln^8^]sodefrin02-1	LC861869	586	192	2								2								
	Cee-[Gln^8^]sodefrin02-2	LC861870	586	192	1								1								
	Cee-[Gln^8^]sodefrin03-1	LC861871	586	192	5					1		4									
	Cee-[Gln^8^]sodefrin04-1	LC861872	316	102	1					1											
	Cee-[Gln^8^]sodefrin05-1	LC861873	586	192	1						1										
	Cee-[Gln^8^]sodefrin05-2	LC861874	586	192	1						1										
	Cee-[Gln^8^]sodefrin06-1	LC861875	586	192	3					2	1										
	Cee-[Gln^8^]sodefrin06-2	LC861876	586	192	2						2										
	Cee-[Gln^8^]sodefrin07-1	LC861877	586	192	1			1													
	Cee-[Gln^8^]sodefrin08-1	LC861878	577	189	1			1										3	1	1	
	Cep-[Gln^8^]sodefrin01-1	LC861897	586	192	5																
	Cep-[Gln^8^]sodefrin02-1	LC861898	625	205	1																1
	**Silefrin precursor**		**529** **–586**	**173** **–192**	**8**									**4**	**2**	**1**	**1**				
	Cep-silefrin01-1	LC861899	586	192	4									4							
	Cep-silefrin02-1	LC861900	586	192	1												1				
	Cep-silefrin03-1	LC861901	586	192	1											1					
	Cep-silefrin04-1	LC861902	529	192	1										1						
	Cep-silefrin05-1	LC861903	586	173	1										1						
**Sodefrin precursor-like factor (SPF)**	**SPF-ß subgroup**		**374** **–539**	**124** **–179**	**73**	**2**	**2**	**3**	**3**	**2**	**3**	**4**	**4**	**4**	**6**	**7**	**7**	**5**	**7**	**7**	**7**
	Cee-SPF-ß01-1	LC861887	506	168	6							3	3								
	Cee-SPF-ß02-1	LC861888	491	163	1								1								
	Cee-SPF-ß03-1	LC861889	506	168	4																
	Cee-SPF-ß04-1	LC861890	491	163	1					1	3										
	Cee-SPF-ß05-1	LC861891	506	168	1					1		1									
	Cee-SPF-ß06-1	LC861892	506	168	4		1	3													
	Cee-SPF-ß07-1	LC861893	506	168	3	1			2												
	Cee-SPF-ß08-1	LC861894	506	168	1		1														
	Cee-SPF-ß09-1	LC861895	506	168	1				1												
	Cee-SPF-ß10-1	LC861896	539	179	1	1															
	Cep-SPF-ß01-1	LC861904	506	168	27									3	1	5	6		4	3	5
	Cep-SPF-ß02-1	LC861905	389	129	1																1
	Cep-SPF-ß03-1	LC861906	506	168	1														1		
	Cep-SPF-ß04-1	LC861907	506	168	1															1	
	Cep-SPF-ß05-1	LC861908	506	168	1														1		
	Cep-SPF-ß06-1	LC861909	506	168	2													2			
	Cep-SPF-ß07-1	LC861910	506	168	1									1							
	Cep-SPF-ß08-1	LC861911	389	129	1																1
	Cep-SPF-ß09-1	LC861912	539	179	6										3	1				2	
	Cep-SPF-ß09-2	LC861913	539	179	1										1						
	Cep-SPF-ß09-3	LC861914	539	179	1											1					
	Cep-SPF-ß10-1	LC861915	539	179	4										1		1		1	1	
	Cep-SPF-ß10-2	LC861916	539	179	3													3			

**Table A2:** List of transcripts encoding sodefrin and sodefrin-variant precursor (S-SVP) and sodefrin precursor-like factor (SPF) that were found to be expressed in *Cynops pyrrhogaster*.

Group	Abbreviated Transcript Name	Accession	mRNA Length (Base)	Number of Amino Acid Residues	Total Number of Clones	Number of Clones per Individual							
						*Cynops pyrrhogaster*							
						A	B	C	D	E	F	G	H
**Sodefrin and Sodefrin-variant Precursor (S-SVP)**	**S-SVP subgroup**		**460** **–577**	**150** **–189**	**52**	**4**	**8**	**5**	**7**	**8**	**7**	**7**	**6**
	**Sodefrin precursor**		**460** **–577**	**150** **–189**	**28**	**4**	**5**	**1**	**3**	**5**	**3**	**4**	**3**
	Cp-sodefrin01-1	LC861931	577	189	6	1	3	1			1		
	Cp-sodefrin01-2	LC861932	577	189	1		1						
	Cp-sodefrin02-1	LC861933	577	189	1		1						
	Cp-sodefrin03-1	LC861934	460	150	1						1		
	Cp-sodefrin04-1	LC861935	577	189	1				1				
	Cp-sodefrin05-1	LC861936	577	189	1							1	
	Cp-sodefrin06-1	LC861937	529	173	1				1				
	Cp-sodefrin07-1	LC861938	529	173	1					1			
	Cp-sodefrin08-1	LC861939	577	189	1	1							
	Cp-sodefrin09-1	LC861940	577	189	1							1	
	Cp-sodefrin10-1	LC861941	529	173	4	2				2			
	Cp-sodefrin10-2	LC861942	529	173	2				1			1	
	Cp-sodefrin11-1	LC861943	514	168	1							1	
	Cp-sodefrin12-1	LC861944	577	189	1					1			
	Cp-sodefrin13-1	LC861945	529	173	1								1
	Cp-sodefrin14-1	LC861946	529	173	1								1
	Cp-sodefrin15-1	LC861947	553	181	1								1
	Cp-sodefrin16-1	LC861948	529	173	1					1			
	Cp-sodefrin17-1	LC861949	529	173	1						1		
	**[Asn^10^]sodefrin precursor**		**577**	**189**	**24**		**3**	**4**	**4**	**3**	**4**	**3**	**3**
	Cp-[Asn^10^]sodefrin01-1	LC861917	577	189	5			3		1		1	
	Cp-[Asn^10^]sodefrin01-2	LC861918	577	189	1							1	
	Cp-[Asn^10^]sodefrin02-1	LC861919	577	189	1					1			
	Cp-[Asn^10^]sodefrin03-1	LC861920	577	189	1					1			
	Cp-[Asn^10^]sodefrin04-1	LC861921	577	189	1			1					
	Cp-[Asn^10^]sodefrin05-1	LC861922	577	189	1						1		
	Cp-[Asn^10^]sodefrin05-2	LC861923	577	189	1						1		
	Cp-[Asn^10^]sodefrin06-1	LC861924	577	189	1								1
	Cp-[Asn^10^]sodefrin07-1	LC861925	577	189	4				2				2
	Cp-[Asn^10^]sodefrin08-1	LC861926	577	189	1		1						
	Cp-[Asn^10^]sodefrin09-1	LC861927	577	189	3				1		2		
	Cp-[Asn^10^]sodefrin09-2	LC861928	577	189	2		2						
	Cp-[Asn^10^]sodefrin10-1	LC861929	577	189	1							1	
	Cp-[Asn^10^]sodefrin11-1	LC861930	577	189	1				1				
**Sodefrin precursor-like factor (SPF)**	**SPF-ß subgroup**		**422** **–539**	**140** **–179**	**12**	**4**		**3**	**1**		**1**	**1**	**2**
	Cp-SPF-ß01-1	LC861950	539	179	2	1							1
	Cp-SPF-ß02-1	LC861951	539	179	1	1							
	Cp-SPF-ß03-1	LC861952	539	179	1	1							
	Cp-SPF-ß04-1	LC861953	539	179	1	1							
	Cp-SPF-ß05-1	LC861954	422	140	1						1		
	Cp-SPF-ß06-1	LC861955	539	179	1								1
	Cp-SPF-ß07-1	LC861956	506	168	1							1	
	Cp-SPF-ß08-1	LC861957	506	168	1			1					
	Cp-SPF-ß09-1	LC861958	506	168	1			1					
	Cp-SPF-ß10-1	LC861959	506	168	1				1				
	Cp-SPF-ß11-1	LC861960	506	168	1			1					
