# Supplementary material for: Genetic Diversity of Sodefrin-Variant Pheromones and Pheromone Responsiveness in Subspecies of the Japanese Sword-Tail Newt Cynops ensicauda
Source: Animals (Basel). 2025 Mar 26;15(7):947. doi: 10.3390/ani15070947 (PMC11987980; doi:10.3390/ani15070947)
Supplement: Supplementary file 1 [file animals-15-00947-s001.zip › animals-3510112-supplementary.pdf]

Table S1. List of the deduced sodefrin and its variant precursor (S-SVP) and sodefrin precursor-like factor (SPF)

Group	Taxon	Species	Name of the Protein Registered	Protein ID	Accession
S-SVP	Salamandridae	*Cynops pyrrhogaster*	sodefrin precursor (sodefrin)	CAB53093.1	AJ245955.1
			aonirin precursor (aonirin: [Val8] sodefrin)	BAF36045.1	AB280579.1
			sodefrin precursor beta sodefrin isoform 01, partial (sodefrin)	AIT39120.1	KM463778.1
			sodefrin precursor beta sodefrin isoform 02, partial (sodefrin)	AIT39121.1	KM463779.1
			sodefrin precursor-like factor beta sodefrin isoform 15, partial ([Asn10] sodefrin)	AIT39134.1	KM463792.1
			sodefrin precursor-like factor ([Asn10] sodefrin)	AMO51434.1	KU213617.1
			sodefrin precursor-like factor ([Asn10] sodefrin)	AMO51436.1	KU213619.1
		C. ensicauda	sodefrin-like peptide precursor (silefrin: [Lue3, Gln8] sodefrin)	CAB53094.1	AJ245956.1
		Lissotriton helveticus	sodefrin precursor-like factor 26 ([His1, Ala3, Pro9]sodefrin)	AJF36152.1	KJ402351.1
		Triturus carnifex	sodefrin-like protein precursor ([Cys1, Thr2, Glu10]sodefrin)	AAL39123.1	AF446080.1
SPF	Salamandridae	*Cynops pyrrhogaster*	sodefrin precursor-like factor beta isoform 03, partial	AIT39122.1	KM463780.1
			sodefrin precursor-like factor beta isoform 04, partial	AIT39123.1	KM463781.1
			sodefrin precursor-like factor beta isoform 05, partial	AIT39124.1	KM463782.1
			sodefrin precursor-like factor beta isoform 06, partial	AIT39125.1	KM463783.1
			sodefrin precursor-like factor beta isoform 07 SS, partial	AIT39126.1	KM463784.1
			sodefrin precursor-like factor beta isoform 08 SS, partial	AIT39127.1	KM463785.1
			sodefrin precursor-like factor beta isoform 09, partial	AIT39128.1	KM463786.1
			sodefrin precursor-like factor beta isoform 10, partial	AIT39129.1	KM463787.1
			sodefrin precursor-like factor beta isoform 11, partial	AIT39130.1	KM463788.1
			sodefrin precursor-like factor beta isoform 12, partial	AIT39131.1	KM463789.1
			sodefrin precursor-like factor beta isoform 13, partial	AIT39132.1	KM463790.1
			sodefrin precursor-like factor beta isoform 14, partial	AIT39133.1	KM463791.1
			sodefrin precursor-like factor alpha isoform 16, partial	AIT39135.1	KM463793.1
			sodefrin precursor-like factor alpha isoform 17, partial	AIT39136.1	KM463794.1
			sodefrin precursor-like factor alpha isoform 18, partial	AIT39137.1	KM463795.1
			sodefrin precursor-like factor alpha isoform 19, partial	AIT39138.1	KM463796.1
			sodefrin precursor-like factor alpha isoform 20, partial	AIT39139.1	KM463797.1
			sodefrin precursor-like factor alpha isoform 21, partial	AIT39140.1	KM463798.1
			sodefrin precursor-like factor alpha isoform 22, partial	AIT39141.1	KM463799.1
			sodefrin precursor-like factor alpha isoform 23, partial	AIT39142.1	KM463800.1
			sodefrin precursor-like factor alpha isoform 24, partial	AIT39143.1	KM463801.1
			sodefrin precursor-like factor alpha isoform 25, partial	AIT39144.1	KM463802.1
			sodefrin precursor-like factor alpha isoform 26, partial	AIT39145.1	KM463803.1
			sodefrin precursor-like factor alpha isoform 27, partial	AIT39146.1	KM463804.1
			sodefrin precursor-like factor alpha isoform 28, partial	AIT39147.1	KM463805.1
			sodefrin precursor-like factor alpha isoform 29, partial	AIT39148.1	KM463806.1
			sodefrin precursor-like factor alpha isoform 30, partial	AIT39149.1	KM463807.1

Group	Taxon	Species	Name of the Protein Registered	Protein ID	Accession
**SPF**	**Salamandridae**	*C. pyrrhogaster*	sodefrin precursor-like factor alpha isoform 31, partial	AIT39150.1	KM463808.1
			sodefrin precursor-like factor alpha isoform 32, partial	AIT39151.1	KM463809.1
			sodefrin precursor-like factor alpha isoform 33, partial	AIT39152.1	KM463810.1
			sodefrin precursor-like factor alpha isoform 34, partial	AIT39153.1	KM463811.1
			sodefrin precursor-like factor	AMO51432.1	KU213615.1
			sodefrin precursor-like factor	AMO51433.1	KU213616.1
			sodefrin precursor-like factor	AMO51435.1	KU213618.1
			sodefrin precursor-like factor	AMO51437.1	KU213620.1
			sodefrin precursor-like factor	AMO51438.1	KU213621.1
			sodefrin precursor-like factor	AMO51439.1	KU213622.1
			sodefrin precursor-like factor	AMO51440.1	KU213623.1
			sodefrin precursor-like factor	AMO51441.1	KU213624.1
			sodefrin precursor-like factor	AMO51442.1	KU213625.1
			sodefrin precursor-like factor	AMO51443.1	KU213626.1
			sodefrin precursor-like factor	AMO51444.1	KU213627.1
			sodefrin precursor-like factor	AMO51445.1	KU213628.1
			sodefrin precursor-like factor	AMO51446.1	KU213629.1
			sodefrin precursor-like factor	AMO51447.1	KU213630.1
			sodefrin precursor-like factor	AMO51448.1	KU213631.1
			sodefrin precursor-like factor	AMO51449.1	KU213632.1
			sodefrin precursor-like factor	AMO51450.1	KU213633.1
			sodefrin precursor-like factor	AMO51451.1	KU213634.1
			sodefrin precursor-like factor	AMO51452.1	KU213635.1
			sodefrin precursor-like factor	AMO51453.1	KU213636.1
			sodefrin precursor-like factor	AMO51454.1	KU213637.1
			sodefrin precursor-like factor	AMO51455.1	KU213638.1
			sodefrin precursor-like factor	AMO51456.1	KU213639.1
		Ichthyosaura alpestris	sodefrin precursor-like factor beta isoform 01, partial	AIT39154.1	KM463812.1
			sodefrin precursor-like factor beta isoform 02, partial	AIT39155.1	KM463813.1
			sodefrin precursor-like factor beta isoform 03, partial	AIT39156.1	KM463814.1
			sodefrin precursor-like factor beta isoform 04, partial	AIT39157.1	KM463815.1
			sodefrin precursor-like factor beta isoform 05, partial	AIT39158.1	KM463816.1
			sodefrin precursor-like factor beta isoform 06, partial	AIT39159.1	KM463817.1
			sodefrin precursor-like factor beta isoform 07, partial	AIT39160.1	KM463818.1
			sodefrin precursor-like factor beta isoform 08, partial	AIT39161.1	KM463819.1
			sodefrin precursor-like factor alpha isoform 09, partial	AIT39162.1	KM463820.1
			sodefrin precursor-like factor alpha isoform 10, partial	AIT39163.1	KM463821.1
			sodefrin precursor-like factor beta isoform 01, partial	AIT39164.1	KM463822.1

Group	Taxon	Species	Name of the Protein Registered	Protein ID	Accession
**SPF**	**Salamandridae**	*I. alpestris*	sodefrin precursor-like factor	AKH13997.1	KP849562.1
			sodefrin precursor-like factor	AKH13998.1	KP849563.1
			sodefrin precursor-like factor	AKH13999.1	KP849564.1
			sodefrin precursor-like factor	AKH14000.1	KP849565.1
			sodefrin precursor-like factor	AKH14001.1	KP849566.1
			sodefrin precursor-like factor	AKH14002.1	KP849567.1
			sodefrin precursor-like factor	AKH14003.1	KP849568.1
			sodefrin precursor-like factor	AKH14004.1	KP849569.1
			sodefrin precursor-like factor	AKH14005.1	KP849570.1
			sodefrin precursor-like factor	AKH14006.1	KP849571.1
			sodefrin precursor-like factor	AKH14007.1	KP849572.1
			sodefrin precursor-like factor	AKH14008.1	KP849573.1
			sodefrin precursor-like factor	AKH14009.1	KP849574.1
			sodefrin precursor-like factor	AKH14010.1	KP849575.1
			sodefrin precursor-like factor	AKH14011.1	KP849576.1
			sodefrin precursor-like factor	AKH14012.1	KP849577.1
			sodefrin precursor-like factor	AKH14013.1	KP849578.1
			sodefrin precursor-like factor	AKH14014.1	KP849579.1
			sodefrin precursor-like factor	AKH14015.1	KP849580.1
			sodefrin precursor-like factor	AKH14016.1	KP849581.1
			sodefrin precursor-like factor	AKH14017.1	KP849582.1
			sodefrin precursor-like factor	AKH14018.1	KP849583.1
			sodefrin precursor-like factor	AKH14019.1	KP849584.1
			sodefrin precursor-like factor	AKH14020.1	KP849585.1
			sodefrin precursor-like factor	AKH14021.1	KP849586.1
			sodefrin precursor-like factor	AKH14022.1	KP849587.1
			sodefrin precursor-like factor	AKH14023.1	KP849588.1
			sodefrin precursor-like factor	AKH14024.1	KP849589.1
		Lissotriton helveticus	sodefrin precursor-like factor 1	AJF36132.1	KJ402326.1
			sodefrin precursor-like factor 2	AJF36133.1	KJ402327.1
			sodefrin precursor-like factor 3	AJF36134.1	KJ402328.1
			sodefrin precursor-like factor 4	AJF36135.1	KJ402329.1
			sodefrin precursor-like factor 5	AJF36136.1	KJ402330.1
			sodefrin precursor-like factor 6	AJF36137.1	KJ402331.1
			sodefrin precursor-like factor 7	AJF36138.1	KJ402332.1
			sodefrin precursor-like factor 8	AJF36139.1	KJ402333.1
			sodefrin precursor-like factor 9	AJF36140.1	KJ402334.1
			sodefrin precursor-like factor 10	AJF36141.1	KJ402335.1

Group	Taxon	Species	Name of the Protein Registered	Protein ID	Accession
**SPF**	**Salamandridae**	*L. helveticus*	sodefrin precursor-like factor 12	AJF36142.1	KJ402337.1
			sodefrin precursor-like factor 13	AJF36143.1	KJ402338.1
			sodefrin precursor-like factor 15	AJF36144.1	KJ402340.1
			sodefrin precursor-like factor 16	AJF36145.1	KJ402341.1
			sodefrin precursor-like factor 18	AJF36146.1	KJ402343.1
			sodefrin precursor-like factor 19	AJF36147.1	KJ402344.1
			sodefrin precursor-like factor 20	AJF36148.1	KJ402345.1
			sodefrin precursor-like factor 21	AJF36149.1	KJ402346.1
			sodefrin precursor-like factor 23	AJF36150.1	KJ402348.1
			sodefrin precursor-like factor 24	AJF36151.1	KJ402349.1
			sodefrin precursor-like factor 28	AJF36153.1	KJ402353.1
			sodefrin precursor-like factor 29	AJF36154.1	KJ402354.1
			sodefrin precursor-like factor 30	AJF36155.1	KJ402355.1
			sodefrin precursor-like factor 31	AJF36156.1	KJ402356.1
			sodefrin precursor-like factor 32	AJF36157.1	KJ402357.1
			sodefrin precursor-like factor beta isoform 01	AIT39186.1	KM463847.1
			sodefrin precursor-like factor beta isoform 02, partial	AIT39187.1	KM463848.1
			sodefrin precursor-like factor beta isoform 03, partial	AIT39188.1	KM463849.1
			sodefrin precursor-like factor beta isoform 04, partial	AIT39189.1	KM463850.1
			sodefrin precursor-like factor beta isoform 05, partial	AIT39190.1	KM463851.1
			sodefrin precursor-like factor beta isoform 06, partial	AIT39191.1	KM463852.1
			sodefrin precursor-like factor beta isoform 07, partial	AIT39192.1	KM463853.1
			sodefrin precursor-like factor beta isoform 08, partial	AIT39193.1	KM463854.1
			sodefrin precursor-like factor beta isoform 09, partial	AIT39194.1	KM463855.1
			sodefrin precursor-like factor beta isoform 10, partial	AIT39195.1	KM463856.1
			sodefrin precursor-like factor beta isoform 11, partial	AIT39196.1	KM463857.1
			sodefrin precursor-like factor beta isoform 12, partial	AIT39197.1	KM463858.1
			sodefrin precursor-like factor beta isoform 13, partial	AIT39198.1	KM463859.1
			sodefrin precursor-like factor beta isoform 14, partial	AIT39199.1	KM463860.1
			sodefrin precursor-like factor beta isoform 15, partial	AIT39200.1	KM463861.1
			sodefrin precursor-like factor beta isoform 16, partial	AIT39201.1	KM463862.1
			sodefrin precursor-like factor beta isoform 17, partial	AIT39202.1	KM463863.1
			sodefrin precursor-like factor beta isoform 18, partial	AIT39203.1	KM463864.1
			sodefrin precursor-like factor beta isoform 19, partial	AIT39204.1	KM463865.1
			sodefrin precursor-like factor alpha isoform 20, partial	AIT39205.1	KM463866.1
			sodefrin precursor-like factor alpha isoform 21, partial	AIT39206.1	KM463867.1
		L. montandoni	sodefrin-like protein precursor	ACB54670.1	EU526845.1
			truncated sodefrin-like protein precursor	ACB54671.1	EU526846.1

Group	Taxon	Species	Name of the Protein Registered	Protein ID	Accession
**SPF**	**Salamandridae**	*L. montandoni*	sodefrin-like protein precursor	ACB54672.1	EU526847.1
		L. vulgaris	sodefrin-like protein precursor	ACB54665.1	EU526840.1
			sodefrin-like protein precursor	ACB54666.1	EU526841.1
			sodefrin-like protein precursor	ACB54667.1	EU526842.1
			sodefrin-like protein precursor	ACB54668.1	EU526843.1
			sodefrin-like protein precursor	ACB54669.1	EU526844.1
			sodefrin precursor-like factor beta isoform 01, partial	AIT39165.1	KM463823.1
			sodefrin precursor-like factor beta isoform 02, partial	AIT39166.1	KM463824.1
			sodefrin precursor-like factor beta isoform 03, partial	AIT39167.1	KM463825.1
			sodefrin precursor-like factor beta isoform 04, partial	AIT39168.1	KM463827.1
			sodefrin precursor-like factor beta isoform 05, partial	AIT39169.1	KM463828.1
			sodefrin precursor-like factor beta isoform 07, partial	AIT39170.1	KM463830.1
			sodefrin precursor-like factor beta isoform 08, partial	AIT39171.1	KM463831.1
			sodefrin precursor-like factor beta isoform 09, partial	AIT39172.1	KM463832.1
			sodefrin precursor-like factor beta isoform 10, partial	AIT39173.1	KM463833.1
			sodefrin precursor-like factor beta isoform 11, partial	AIT39174.1	KM463834.1
			sodefrin precursor-like factor beta isoform 12, partial	AIT39175.1	KM463835.1
			sodefrin precursor-like factor beta isoform 13	AIT39176.1	KM463836.1
			sodefrin precursor-like factor alpha isoform 14, partial	AIT39177.1	KM463837.1
			sodefrin precursor-like factor alpha isoform 15, partial	AIT39178.1	KM463838.1
			sodefrin precursor-like factor alpha isoform 16, partial	AIT39179.1	KM463839.1
			sodefrin precursor-like factor alpha isoform 17, partial	AIT39180.1	KM463840.1
			sodefrin precursor-like factor alpha isoform 18, partial	AIT39181.1	KM463841.1
			sodefrin precursor-like factor alpha isoform 19, partial	AIT39182.1	KM463842.1
			sodefrin precursor-like factor beta isoform 01, partial	AIT39183.1	KM463843.1
			sodefrin precursor-like factor beta isoform 02, partial	AIT39184.1	KM463844.1
			sodefrin precursor-like factor beta isoform 03, partial	AIT39185.1	KM463845.1
		Notophthalmus viridescens	sodefrin precursor-like factor beta isoform 01, partial	AIT39207.1	KM463868.1
			sodefrin precursor-like factor beta isoform 02, partial	AIT39208.1	KM463869.1
			sodefrin precursor-like factor beta isoform 03, partial	AIT39209.1	KM463870.1
			sodefrin precursor-like factor beta isoform 04, partial	AIT39210.1	KM463871.1
			sodefrin precursor-like factor beta isoform 05, partial	AIT39211.1	KM463872.1
			sodefrin precursor-like factor beta isoform 06, partial	AIT39212.1	KM463873.1
			sodefrin precursor-like factor beta isoform 07, partial	AIT39213.1	KM463874.1
			sodefrin precursor-like factor beta isoform 08, partial	AIT39214.1	KM463875.1
			sodefrin precursor-like factor beta isoform 09, partial	AIT39215.1	KM463876.1
			sodefrin precursor-like factor beta isoform 10, partial	AIT39216.1	KM463877.1
			sodefrin precursor-like factor beta isoform 11 SS, partial	AIT39217.1	KM463878.1

Group	Taxon	Species	Name of the Protein Registered	Protein ID	Accession
**SPF**	**Salamandridae**	*N. viridescens*	sodefrin precursor-like factor beta isoform 12, partial	AIT39218.1	KM463879.1
			sodefrin precursor-like factor beta isoform 13, partial	AIT39219.1	KM463880.1
			sodefrin precursor-like factor beta isoform 14, partial	AIT39220.1	KM463881.1
			sodefrin precursor-like factor beta isoform 15, partial	AIT39221.1	KM463882.1
			sodefrin precursor-like factor beta isoform 16, partial	AIT39222.1	KM463883.1
			sodefrin precursor-like factor beta isoform 17 SS, partial	AIT39223.1	KM463884.1
			sodefrin precursor-like factor beta isoform 18, partial	AIT39224.1	KM463886.1
			sodefrin precursor-like factor beta isoform 19, partial	AIT39225.1	KM463887.1
			sodefrin precursor-like factor beta isoform 20, partial	AIT39226.1	KM463888.1
			sodefrin precursor-like factor beta isoform 21, partial	AIT39227.1	KM463889.1
			sodefrin precursor-like factor beta isoform 22, partial	AIT39228.1	KM463891.1
			sodefrin precursor-like factor beta isoform 23	AIT39229.1	KM463892.1
			sodefrin precursor-like factor beta isoform 24	AIT39230.1	KM463893.1
			sodefrin precursor-like factor beta isoform 25	AIT39231.1	KM463894.1
			sodefrin precursor-like factor beta isoform 26	AIT39232.1	KM463895.1
			sodefrin precursor-like factor alpha isoform 27, partial	AIT39233.1	KM463896.1
			sodefrin precursor-like factor alpha isoform 28, partial	AIT39234.1	KM463897.1
			sodefrin precursor-like factor alpha isoform 29, partial	AIT39235.1	KM463898.1
			sodefrin precursor-like factor alpha isoform 30, partial	AIT39236.1	KM463899.1
			sodefrin precursor-like factor alpha isoform 31, partial	AIT39237.1	KM463900.1
			sodefrin precursor-like factor alpha isoform 32, partial	AIT39238.1	KM463901.1
			sodefrin precursor-like factor alpha isoform 33, partial	AIT39239.1	KM463902.1
			sodefrin precursor-like factor alpha isoform 34, partial	AIT39240.1	KM463903.1
			sodefrin precursor-like factor alpha isoform 35, partial	AIT39241.1	KM463904.1
			sodefrin precursor-like factor alpha isoform 36, partial	AIT39242.1	KM463905.1
			sodefrin precursor-like factor alpha isoform 37, partial	AIT39243.1	KM463906.1
			sodefrin precursor-like factor alpha isoform 38, partial	AIT39244.1	KM463907.1
			sodefrin precursor-like factor alpha isoform 39, partial	AIT39245.1	KM463908.1
			sodefrin precursor-like factor alpha isoform 40, partial	AIT39246.1	KM463909.1
			sodefrin precursor-like factor alpha isoform 41, partial	AIT39247.1	KM463910.1
			sodefrin precursor-like factor alpha isoform 42, partial	AIT39248.1	KM463911.1
			sodefrin precursor-like factor alpha isoform 43, partial	AIT39249.1	KM463912.1
			sodefrin precursor-like factor alpha isoform 44, partial	AIT39250.1	KM463913.1
			sodefrin precursor-like factor alpha isoform 45, partial	AIT39251.1	KM463914.1
			sodefrin precursor-like factor alpha isoform 46, partial	AIT39252.1	KM463915.1
			sodefrin precursor-like factor alpha isoform 47, partial	AIT39253.1	KM463916.1
			sodefrin precursor-like factor, partial	AKA95426.1	KP118895.1
			sodefrin precursor-like factor	AKA95427.1	KP118896.1

Group	Taxon	Species	Name of the Protein Registered	Protein ID	Accession
**SPF**	**Salamandridae**	*N. viridescens*	sodefrin precursor-like factor	AKA95428.1	KP118897.1
			sodefrin precursor-like factor, partial	AKA95429.1	KP118898.1
			sodefrin precursor-like factor, partial	AKA95430.1	KP118899.1
			sodefrin precursor-like factor	AKA95431.1	KP118900.1
			sodefrin precursor-like factor	AKA95432.1	KP118901.1
			sodefrin precursor-like factor	AKA95433.1	KP118902.1
			sodefrin precursor-like factor	AKA95434.1	KP118903.1
			sodefrin precursor-like factor	AKA95435.1	KP118904.1
			sodefrin precursor-like factor	AKA95436.1	KP118905.1
			sodefrin precursor-like factor	AKA95437.1	KP118906.1
			sodefrin precursor-like factor	AKA95438.1	KP118907.1
			sodefrin precursor-like factor	AKA95439.1	KP118908.1
			sodefrin precursor-like factor	AKA95440.1	KP118909.1
			sodefrin precursor-like factor	AKA95441.1	KP118910.1
			sodefrin precursor-like factor	AKA95442.1	KP118911.1
			sodefrin precursor-like factor	AKA95443.1	KP118912.1
			sodefrin precursor-like factor	AKA95444.1	KP118913.1
			sodefrin precursor-like factor	AKA95445.1	KP118914.1
			sodefrin precursor-like factor	AKA95446.1	KP118915.1
			sodefrin precursor-like factor, partial	AKA95447.1	KP118916.1
			sodefrin precursor-like factor, partial	AKA95448.1	KP118917.1
			sodefrin precursor-like factor	AKA95449.1	KP118918.1
			sodefrin precursor-like factor, partial	AKA95450.1	KP118919.1
			sodefrin precursor-like factor, partial	AKA95451.1	KP118920.1
			sodefrin precursor-like factor, partial	AKA95452.1	KP118921.1
			sodefrin precursor-like factor, partial	AKA95453.1	KP118922.1
			sodefrin precursor-like factor, partial	AKA95454.1	KP118923.1
			sodefrin precursor-like factor, partial	AKA95455.1	KP118924.1
			sodefrin precursor-like factor, partial	AKA95456.1	KP118925.1
			sodefrin precursor-like factor, partial	AKA95457.1	KP118926.1
			sodefrin precursor-like factor, partial	AKA95458.1	KP118927.1
			sodefrin precursor-like factor, partial	AKA95459.1	KP118928.1
			sodefrin precursor-like factor	AKA95460.1	KP118929.1
			sodefrin precursor-like factor	AKA95461.1	KP118930.1
			sodefrin precursor-like factor	AKA95462.1	KP118931.1
			sodefrin precursor-like factor	AKA95463.1	KP118932.1
			sodefrin precursor-like factor	AKA95464.1	KP118933.1
			sodefrin precursor-like factor	AKA95465.1	KP118934.1

Group	Taxon	Species	Name of the Protein Registered	Protein ID	Accession
**SPF**	**Salamandridae**	*N. viridescens*	sodefrin precursor-like factor	AKA95466.1	KP118935.1
			sodefrin precursor-like factor	AKA95467.1	KP118936.1
			sodefrin precursor-like factor	AKA95468.1	KP118937.1
			sodefrin precursor-like factor	AKA95469.1	KP118938.1
			sodefrin precursor-like factor	AKA95470.1	KP118939.1
			sodefrin precursor-like factor, partial	AKA95471.1	KP118940.1
			sodefrin precursor-like factor, partial	AKA95472.1	KP118941.1
			sodefrin precursor-like factor	AKA95473.1	KP118943.1
			sodefrin precursor-like factor	AKA95474.1	KP118944.1
			sodefrin precursor-like factor	AKA95475.1	KP118945.1
			sodefrin precursor-like factor	AKA95476.1	KP118946.1
			sodefrin precursor-like factor, partial	AKA95477.1	KP118947.1
			sodefrin precursor-like factor	AKA95478.1	KP118948.1
			sodefrin precursor-like factor	AKA95479.1	KP118949.1
			sodefrin precursor-like factor, partial	AKA95480.1	KP118950.1
			sodefrin precursor-like factor, partial	AKA95481.1	KP118951.1
			sodefrin precursor-like factor, partial	AKA95482.1	KP118952.1
			sodefrin precursor-like factor, partial	AKA95483.1	KP118953.1
			sodefrin precursor-like factor, partial	AKA95484.1	KP118954.1
			sodefrin precursor-like factor, partial	AKA95485.1	KP118955.1
		Pachytriton granulosus	sodefrin precursor-like factor beta isoform 01, partial	AIT39111.1	KM463769.1
			sodefrin precursor-like factor beta isoform 02, partial	AIT39112.1	KM463770.1
			sodefrin precursor-like factor beta isoform 03, partial	AIT39113.1	KM463771.1
			sodefrin precursor-like factor beta isoform 04, partial	AIT39114.1	KM463772.1
			sodefrin precursor-like factor beta isoform 05, partial	AIT39115.1	KM463773.1
			sodefrin precursor-like factor beta isoform 06, partial	AIT39116.1	KM463774.1
			sodefrin precursor-like factor beta isoform 07, partial	AIT39117.1	KM463775.1
			sodefrin precursor-like factor alpha isoform 08, partial	AIT39118.1	KM463776.1
			sodefrin precursor-like factor alpha isoform 09, partial	AIT39119.1	KM463777.1
		Pleurodeles waltl	sodefrin precursor-like factor beta isoform 01	AIT39259.1	KM463922.1
			sodefrin precursor-like factor beta isoform 02	AIT39260.1	KM463923.1
			sodefrin precursor-like factor beta isoform 03	AIT39261.1	KM463924.1
			sodefrin precursor-like factor beta isoform 04, partial	AIT39262.1	KM463925.1
			sodefrin precursor-like factor beta isoform 05	AIT39263.1	KM463926.1
			sodefrin precursor-like factor beta isoform 06, partial	AIT39264.1	KM463927.1
			sodefrin precursor-like factor beta isoform 07, partial	AIT39265.1	KM463928.1
			sodefrin precursor-like factor beta isoform 09	AIT39266.1	KM463930.1
			sodefrin precursor-like factor beta isoform 10	AIT39267.1	KM463931.1

Group	Taxon	Species	Name of the Protein Registered	Protein ID	Accession
**SPF**	**Salamandridae**	*P. waltl*	sodefrin precursor-like factor alpha isoform 11, partial	AIT39268.1	KM463932.1
			sodefrin precursor-like factor alpha isoform 12, partial	AIT39269.1	KM463933.1
		Taricha granulosa	sodefrin precursor-like factor beta isoform 01, partial	AIT39254.1	KM463917.1
			sodefrin precursor-like factor beta isoform 02, partial	AIT39255.1	KM463918.1
			sodefrin precursor-like factor alpha isoform 03, partial	AIT39256.1	KM463919.1
			sodefrin precursor-like factor alpha isoform 04, partial	AIT39257.1	KM463920.1
			sodefrin precursor-like factor alpha isoform 05, partial	AIT39258.1	KM463921.1
	**Ambystomatidae**	*Ambystoma mexicanum*	sodefrin precursor-like factor	AME17849.1	KU043451.1
			sodefrin precursor-like factor	AME17850.1	KU043452.1
			sodefrin precursor-like factor	AME17851.1	KU043453.1
			sodefrin precursor-like factor	AME17852.1	KU043454.1
			sodefrin precursor-like factor	AME17853.1	KU043455.1
			sodefrin precursor-like factor	AME17854.1	KU043456.1
			sodefrin precursor-like factor	AME17855.1	KU043457.1
			sodefrin precursor-like factor	AME17856.1	KU043458.1
			sodefrin precursor-like factor	AME17857.1	KU043459.1
			sodefrin precursor-like factor	AME17858.1	KU043460.1
			sodefrin precursor-like factor	AME17859.1	KU043461.1
			sodefrin precursor-like factor	AME17860.1	KU043462.1
			sodefrin precursor-like factor	AME17861.1	KU043463.1
	**Plethodontidae**	*Aneides ferreus*	sodefrin precursor-like factor	AAZ06335.1	DQ097071.1
			sodefrin precursor-like factor	AAZ06336.1	DQ097072.1
		Desmognathus brimleyorum	sodefrin precursor-like factor alpha 1, partial	UVZ00866.1	MZ927630.1
			sodefrin precursor-like factor alpha 2, partial	UVZ00869.1	MZ927633.1
			sodefrin precursor-like factor alpha 3, partial	UVZ00871.1	MZ927635.1
		D. brimleyorum	sodefrin precursor-like factor beta 1, partial	UVZ00874.1	MZ927638.1
			sodefrin precursor-like factor beta 2, partial	UVZ00876.1	MZ927640.1
			sodefrin precursor-like factor beta 4, partial	UVZ00880.1	MZ927644.1
			sodefrin precursor-like factor beta 5, partial	UVZ00881.1	MZ927645.1
		D. monticola	sodefrin precursor-like factor	AAZ06324.1	DQ097060.1
			sodefrin precursor-like factor	AAZ06326.1	DQ097062.1
			sodefrin precursor-like factor	AAZ06327.1	DQ097063.1
			sodefrin precursor-like factor	AAZ06328.1	DQ097064.1
			sodefrin precursor-like factor	AAZ06333.1	DQ097069.1
		D. ocoee	sodefrin precursor-like factor	AAZ06329.1	DQ097065.1
			sodefrin precursor-like factor	AAZ06330.1	DQ097066.1
			sodefrin precursor-like factor	AAZ06332.1	DQ097068.1
			sodefrin precursor-like factor	AAZ06334.1	DQ097070.1

Group	Taxon	Species	Name of the Protein Registered	Protein ID	Accession
**SPF**	**Plethodontidae**	*D. ocoee*	sodefrin precursor-like factor SPF isoform I01, partial	AKN46060.1	KP410906.1
			sodefrin precursor-like factor SPF isoform I02, partial	AKN46061.1	KP410907.1
			sodefrin precursor-like factor SPF isoform I03, partial	AKN46062.1	KP410908.1
			sodefrin precursor-like factor SPF isoform I04, partial	AKN46063.1	KP410909.1
			sodefrin precursor-like factor SPF isoform I05, partial	AKN46064.1	KP410910.1
			sodefrin precursor-like factor SPF isoform I06, partial	AKN46065.1	KP410911.1
			sodefrin precursor-like factor SPF isoform I07, partial	AKN46066.1	KP410912.1
			sodefrin precursor-like factor SPF isoform I08, partial	AKN46067.1	KP410913.1
			sodefrin precursor-like factor SPF isoform I09, partial	AKN46068.1	KP410914.1
			sodefrin precursor-like factor SPF isoform I10, partial	AKN46069.1	KP410915.1
			sodefrin precursor-like factor SPF isoform I11, partial	AKN46070.1	KP410916.1
			sodefrin precursor-like factor SPF isoform I12, partial	AKN46071.1	KP410917.1
			sodefrin precursor-like factor SPF isoform I13, partial	AKN46072.1	KP410918.1
			sodefrin precursor-like factor SPF isoform I14, partial	AKN46073.1	KP410919.1
			sodefrin precursor-like factor SPF isoform I15, partial	AKN46074.1	KP410920.1
			sodefrin precursor-like factor SPF isoform I16, partial	AKN46075.1	KP410921.1
			sodefrin precursor-like factor SPF isoform I17, partial	AKN46076.1	KP410922.1
			sodefrin precursor-like factor SPF isoform I18, partial	AKN46077.1	KP410923.1
			sodefrin precursor-like factor SPF isoform I19, partial	AKN46078.1	KP410924.1
			sodefrin precursor-like factor SPF isoform I20, partial	AKN46079.1	KP410925.1
			sodefrin precursor-like factor SPF isoform I21, partial	AKN46080.1	KP410926.1
			sodefrin precursor-like factor SPF isoform I22, partial	AKN46081.1	KP410927.1
			sodefrin precursor-like factor SPF isoform I23, partial	AKN46082.1	KP410928.1
			sodefrin precursor-like factor SPF isoform I24, partial	AKN46083.1	KP410929.1
			sodefrin precursor-like factor SPF isoform I25, partial	AKN46084.1	KP410930.1
			sodefrin precursor-like factor SPF isoform II01, partial	AKN46085.1	KP410931.1
			sodefrin precursor-like factor SPF isoform II02, partial	AKN46086.1	KP410932.1
			sodefrin precursor-like factor SPF isoform II03, partial	AKN46087.1	KP410933.1
			sodefrin precursor-like factor SPF isoform II04, partial	AKN46088.1	KP410934.1
			sodefrin precursor-like factor SPF isoform II05, partial	AKN46089.1	KP410935.1
			sodefrin precursor-like factor SPF isoform II06, partial	AKN46090.1	KP410936.1
			sodefrin precursor-like factor SPF isoform II07, partial	AKN46091.1	KP410937.1
			sodefrin precursor-like factor SPF isoform II08, partial	AKN46092.1	KP410938.1
			sodefrin precursor-like factor SPF isoform III01, partial	AKN46093.1	KP410939.1
			sodefrin precursor-like factor SPF isoform III02, partial	AKN46094.1	KP410940.1
			sodefrin precursor-like factor SPF isoform III03, partial	AKN46095.1	KP410941.1
			sodefrin precursor-like factor SPF isoform III04, partial	AKN46096.1	KP410942.1
			sodefrin precursor-like factor SPF isoform III05, partial	AKN46097.1	KP410943.1

Group	Taxon	Species	Name of the Protein Registered	Protein ID	Accession
**SPF**	**Plethodontidae**	*D. ocoee*	sodefrin precursor-like factor SPF isoform III06, partial	AKN46098.1	KP410944.1
			sodefrin precursor-like factor SPF isoform III07, partial	AKN46099.1	KP410945.1
			sodefrin precursor-like factor SPF isoform III08, partial	AKN46100.1	KP410946.1
			sodefrin precursor-like factor SPF isoform IV01, partial	AKN46101.1	KP410947.1
			sodefrin precursor-like factor SPF isoform IV02, partial	AKN46102.1	KP410948.1
			sodefrin precursor-like factor SPF isoform IV03, partial	AKN46103.1	KP410949.1
			sodefrin precursor-like factor SPF isoform IV04, partial	AKN46104.1	KP410950.1
			sodefrin precursor-like factor SPF isoform IV05, partial	AKN46105.1	KP410951.1
			sodefrin precursor-like factor SPF isoform V01, partial	AKN46106.1	KP410952.1
			sodefrin precursor-like factor SPF isoform VI01, partial	AKN46107.1	KP410953.1
			sodefrin precursor-like factor SPF isoform VI02, partial	AKN46108.1	KP410954.1
			sodefrin precursor-like factor SPF isoform VI03, partial	AKN46109.1	KP410955.1
			sodefrin precursor-like factor SPF isoform VI04, partial	AKN46110.1	KP410956.1
			sodefrin precursor-like factor SPF isoform VI05, partial	AKN46111.1	KP410957.1
			sodefrin precursor-like factor SPF isoform VI06, partial	AKN46112.1	KP410958.1
			sodefrin precursor-like factor SPF isoform VI07, partial	AKN46113.1	KP410959.1
			sodefrin precursor-like factor SPF isoform VI08, partial	AKN46114.1	KP410960.1
			sodefrin precursor-like factor SPF isoform VI09, partial	AKN46115.1	KP410961.1
			sodefrin precursor-like factor SPF isoform VI10, partial	AKN46116.1	KP410962.1
			sodefrin precursor-like factor SPF isoform VI11, partial	AKN46117.1	KP410963.1
			sodefrin precursor-like factor SPF isoform VI12, partial	AKN46118.1	KP410964.1
			sodefrin precursor-like factor SPF isoform VI13, partial	AKN46119.1	KP410965.1
			sodefrin precursor-like factor SPF hybrid isoform i-II01, partial	AKN46120.1	KP410966.1
			sodefrin precursor-like factor SPF hybrid isoform i-II02, partial	AKN46121.1	KP410967.1
			sodefrin precursor-like factor SPF hybrid isoform II-I01, partial	AKN46122.1	KP410968.1
			sodefrin precursor-like factor SPF hybrid isoform II-I02, partial	AKN46123.1	KP410969.1
			sodefrin precursor-like factor SPF hybrid isoform i-II03, partial	AKN46124.1	KP410970.1
			sodefrin precursor-like factor SPF hybrid isoform III-II01, partial	AKN46125.1	KP410971.1
			sodefrin precursor-like factor SPF hybrid isoform III-II02, partial	AKN46126.1	KP410972.1
			sodefrin precursor-like factor SPF hybrid isoform II-i-II01, partial	AKN46127.1	KP410973.1
			sodefrin precursor-like factor SPF hybrid isoform v-II01, partial	AKN46128.1	KP410974.1
			sodefrin precursor-like factor SPF hybrid isoform IV-I01, partial	AKN46129.1	KP410975.1
			sodefrin precursor-like factor SPF hybrid isoform IV-I02, partial	AKN46130.1	KP410976.1
			sodefrin precursor-like factor SPF hybrid isoform IV-II01, partial	AKN46131.1	KP410977.1
			sodefrin precursor-like factor SPF hybrid isoform i-III-II01, partial	AKN46132.1	KP410978.1
			sodefrin precursor-like factor SPF hybrid isoform i-IV01, partial	AKN46133.1	KP410979.1
			sodefrin precursor-like factor SPF hybrid isoform II-III01, partial	AKN46134.1	KP410980.1
		Eurycea guttolineata	sodefrin precursor-like factor	AAZ06338.1	DQ097074.1

Group	Taxon	Species	Name of the Protein Registered	Protein ID	Accession
**SPF**	**Plethodontidae**	*E. tynerensis*	sodefrin precursor-like factor alpha 1, partial	UVZ00865.1	MZ927629.1
			sodefrin precursor-like factor alpha 2	UVZ00868.1	MZ927632.1
			sodefrin precursor-like factor alpha 3	UVZ00870.1	MZ927634.1
			sodefrin precursor-like factor beta 1	UVZ00873.1	MZ927637.1
			sodefrin precursor-like factor beta 2	UVZ00875.1	MZ927639.1
			sodefrin precursor-like factor beta 3	UVZ00878.1	MZ927642.1
			sodefrin precursor-like factor beta 4	UVZ00879.1	MZ927643.1
		E. wilderae	sodefrin precursor-like factor	AAZ06337.1	DQ097073.1
		Plethodon albagula	sodefrin precursor-like factor alpha 1, partial	UVZ00867.1	MZ927631.1
			sodefrin precursor-like factor alpha 3, partial	UVZ00872.1	MZ927636.1
			sodefrin precursor-like factor beta 2, partial	UVZ00877.1	MZ927641.1
			sodefrin precursor-like factor beta 5	UVZ00882.1	MZ927646.1
		P. aureolus	sodefrin precursor-like protein	ABD34636.1	DQ384552.1
			sodefrin precursor-like protein	ABD34637.1	DQ384553.1
			sodefrin precursor-like protein	ABD34638.1	DQ384554.1
			sodefrin precursor-like protein	ABD34641.1	DQ384557.1
			sodefrin precursor-like protein	ABD34644.1	DQ384560.1
		P. chattahoochee	sodefrin precursor-like protein	ABD34635.1	DQ384551.1
			sodefrin precursor-like protein	ABD34639.1	DQ384555.1
			sodefrin precursor-like protein	ABD34646.1	DQ384562.1
			sodefrin precursor-like protein	ABD34650.1	DQ384566.1
			sodefrin precursor-like protein	ABD34652.1	DQ384568.1
			sodefrin precursor-like protein	ABD34653.1	DQ384569.1
			sodefrin precursor-like protein	ABD34654.1	DQ384570.1
		P. cheoah	sodefrin precursor-like factor	AAZ06296.1	DQ097032.1
			sodefrin precursor-like factor	AAZ06300.1	DQ097036.1
			sodefrin precursor-like factor	AAZ06310.1	DQ097046.1
			sodefrin precursor-like factor	AAZ06319.1	DQ097055.1
		P. cinereus	sodefrin precursor-like factor	AAZ06281.1	DQ097017.1
			sodefrin precursor-like factor	AAZ06282.1	DQ097018.1
			sodefrin precursor-like factor	AAZ06284.1	DQ097020.1
		P. cylindraceus	sodefrin precursor-like protein	ABD34651.1	DQ384567.1
		P. dorsalis	sodefrin precursor-like protein	ABD34632.1	DQ384548.1
		P. hoffmani	sodefrin precursor-like protein	ABD34621.1	DQ384537.1
			sodefrin precursor-like protein	ABD34622.1	DQ384538.1
		P. jordani	sodefrin precursor-like factor	AAZ06299.1	DQ097035.1
			sodefrin precursor-like factor	AAZ06306.1	DQ097042.1
			sodefrin precursor-like factor	AAZ06317.1	DQ097053.1

Group	Taxon	Species	Name of the Protein Registered	Protein ID	Accession
**SPF**	**Plethodontidae**	*P. jordani*	sodefrin precursor-like factor	AAZ06322.1	DQ097058.1
		P. kentucki	sodefrin precursor-like protein	ABD34648.1	DQ384564.1
			sodefrin precursor-like protein	ABD34649.1	DQ384565.1
		P. metcalfi	sodefrin precursor-like factor	AAZ06298.1	DQ097034.1
			sodefrin precursor-like factor	AAZ06302.1	DQ097038.1
			sodefrin precursor-like factor	AAZ06315.1	DQ097051.1
			sodefrin precursor-like factor	AAZ06316.1	DQ097052.1
			sodefrin precursor-like factor	AAZ06321.1	DQ097057.1
		P. mississippi	sodefrin precursor-like protein	ABD34634.1	DQ384550.1
			sodefrin precursor-like protein	ABD34640.1	DQ384556.1
			sodefrin precursor-like protein	ABD34642.1	DQ384558.1
			sodefrin precursor-like protein	ABD34643.1	DQ384559.1
			sodefrin precursor-like protein	ABD34645.1	DQ384561.1
			sodefrin precursor-like protein	ABD34647.1	DQ384563.1
		P. montanus	sodefrin precursor-like factor	AAZ06293.1	DQ097029.1
			sodefrin precursor-like factor	AAZ06295.1	DQ097031.1
			sodefrin precursor-like factor	AAZ06314.1	DQ097050.1
		P. ouachitae	sodefrin precursor-like factor	AAZ06307.1	DQ097043.1
			sodefrin precursor-like factor	AAZ06308.1	DQ097044.1
			sodefrin precursor-like factor	AAZ06312.1	DQ097048.1
		P. richmondi	sodefrin precursor-like factor	AAZ06283.1	DQ097019.1
			sodefrin precursor-like factor	AAZ06285.1	DQ097021.1
			sodefrin precursor-like factor	AAZ06286.1	DQ097022.1
			sodefrin precursor-like factor	AAZ06287.1	DQ097023.1
			sodefrin precursor-like factor	AAZ06289.1	DQ097025.1
			sodefrin precursor-like factor	AAZ06290.1	DQ097026.1
		P. serratus	sodefrin precursor-like protein	ABD34620.1	DQ384536.1
		P. shermani	sodefrin precursor-like factor	AAZ06301.1	DQ097037.1
			sodefrin precursor-like factor	AAZ06311.1	DQ097047.1
		P. stormi	sodefrin precursor-like factor	AAZ06325.1	DQ097061.1
			sodefrin precursor-like factor	AAZ06331.1	DQ097067.1
		P. teyahalee	sodefrin precursor-like factor	AAZ06292.1	DQ097028.1
			sodefrin precursor-like factor	AAZ06304.1	DQ097040.1
			sodefrin precursor-like factor	AAZ06309.1	DQ097045.1
			sodefrin precursor-like factor	AAZ06313.1	DQ097049.1
			sodefrin precursor-like factor	AAZ06318.1	DQ097054.1
			sodefrin precursor-like factor	AAZ06320.1	DQ097056.1
			sodefrin precursor-like factor	AAZ06323.1	DQ097059.1

Group	Taxon	Species	Name of the Protein Registered	Protein ID	Accession
SPF	Plethodontidae	*P. ventralis*	sodefrin precursor-like protein	ABD34623.1	DQ384539.1
			sodefrin precursor-like protein	ABD34624.1	DQ384540.1
			sodefrin precursor-like protein	ABD34626.1	DQ384542.1
			sodefrin precursor-like protein	ABD34629.1	DQ384545.1
			sodefrin precursor-like protein	ABD34630.1	DQ384546.1
		P. websteri	sodefrin precursor-like factor	AAZ06280.1	DQ097016.1
			sodefrin precursor-like factor	AAZ06288.1	DQ097024.1
			sodefrin precursor-like factor	AAZ06291.1	DQ097027.1
		P. wehrlei	sodefrin precursor-like factor	AAZ06294.1	DQ097030.1
			sodefrin precursor-like factor	AAZ06297.1	DQ097033.1
			sodefrin precursor-like protein	ABD34631.1	DQ384547.1
			sodefrin precursor-like protein	ABD34633.1	DQ384549.1
			sodefrin precursor-like protein	ABD34625.1	DQ384541.1
			sodefrin precursor-like protein	ABD34627.1	DQ384543.1
			sodefrin precursor-like protein	ABD34628.1	DQ384544.1
		P. yonahlossee	sodefrin precursor-like factor	AAZ06303.1	DQ097039.1
			sodefrin precursor-like factor	AAZ06305.1	DQ097041.1
	Hylidae (Anura)	*Boana cinerascens*	sodefrin precursor-like factor A	QDH44587.1	MK457706.1
			sodefrin precursor-like factor B	QDH44588.1	MK457707.1
			sodefrin precursor-like factor C	QDH44589.1	MK457708.1
			sodefrin precursor-like factor D	QDH44590.1	MK457709.1
			sodefrin precursor-like factor E	QDH44591.1	MK457710.1
			sodefrin precursor-like factor F	QDH44592.1	MK457711.1
		Hyloscirtus phyllognathus	sodefrin precursor-like factor A	QDH44593.1	MK457712.1
			sodefrin precursor-like factor B	QDH44594.1	MK457713.1
			sodefrin precursor-like factor C	QDH44595.1	MK457714.1
			sodefrin precursor-like factor D	QDH44596.1	MK457715.1
			sodefrin precursor-like factor E	QDH44597.1	MK457716.1
			sodefrin precursor-like factor F	QDH44598.1	MK457717.1
		Plectrohyla matudai	sodefrin precursor-like factor A	UGY75778.1	OL598423.1
			sodefrin precursor-like factor B	UGY75779.1	OL598424.1
			sodefrin precursor-like factor C	UGY75780.1	OL598425.1
		P. sagorum	sodefrin precursor-like factor A	UGY75775.1	OL598420.1
			sodefrin precursor-like factor B	UGY75776.1	OL598421.1
			sodefrin precursor-like factor C	UGY75777.1	OL598422.1
	Nyctibatrachidae (Anura)	*Nyctibatrachus humayuni*	sodefrin precursor-like factor A	QDH44600.1	MK457719.1
		N. petraeus	sodefrin precursor-like factor A	QDH44599.1	MK457718.1
	Pipidae (Anura)	*Hymenochirus boettgeri*	sodefrin precursor-like factor A	QDH44586.1	MK457705.1

Group	Taxon	Species	Name of the Protein Registered	Protein ID	Accession
**SPF**	**Ranidae (Anura)**	*Lithobates vaillanti*	sodefrin precursor-like factor A	WCP86177.1	OP533926.1
			sodefrin precursor-like factor B	WCP86178.1	OP533927.1
			sodefrin precursor-like factor C	WCP86179.1	OP533928.1
